# Dense Bicoid hubs accentuate binding along the morphogen gradient

**DOI:** 10.1101/gad.305078.117

**Published:** 2017-09-01

**Authors:** Mustafa Mir, Armando Reimer, Jenna E. Haines, Xiao-Yong Li, Michael Stadler, Hernan Garcia, Michael B. Eisen, Xavier Darzacq

**Affiliations:** 1 Department of Molecular and Cell Biology, University of California at Berkeley, Berkeley, California, 94720, USA;; 2 Biophysics Graduate Group, University of California at Berkeley, Berkeley, California, 94720, USA;; 3 Howard Hughes Medical Institute, University of California at Berkeley, Berkeley, California, 94720, USA;; 4 Department of Physics, University of California at Berkeley, Berkeley, California, 94720, USA;; 5 Department of Integrative Biology, University of California at Berkeley, Berkeley, California, 94720, USA

**Keywords:** Bicoid, Zelda, *Drosophila*, morphogen, single-molecule fluorescence, transcription factor dynamics

## Abstract

Morphogen gradients direct the spatial patterning of developing embryos; however, the mechanisms by which these gradients are interpreted remain elusive. Here we used lattice light-sheet microscopy to perform in vivo single-molecule imaging in early *Drosophila melanogaster* embryos of the transcription factor Bicoid that forms a gradient and initiates patterning along the anteroposterior axis. In contrast to canonical models, we observed that Bicoid binds to DNA with a rapid off rate throughout the embryo such that its average occupancy at target loci is on-rate-dependent. We further observed Bicoid forming transient “hubs” of locally high density that facilitate binding as factor levels drop, including in the posterior, where we observed Bicoid binding despite vanishingly low protein levels. We propose that localized modulation of transcription factor on rates via clustering provides a general mechanism to facilitate binding to low-affinity targets and that this may be a prevalent feature of other developmental transcription factors.

## Abstract (precis)

Mir et al. used lattice light-sheet microscopy to perform in vivo single-molecule imaging in early *Drosophila melanogaster* embryos and observed Bicoid forming transient “hubs” of locally high density that facilitate binding as factor levels drop.

Spatial patterning during embryonic development is orchestrated through concentration gradients of regulatory molecules known as morphogens (Turing 1952; Wolpert 1969). The maternally deposited transcription factor (TF) Bicoid (BCD) in *Drosophila melanogaster* was the first identified morphogen (Driever and Nusslein-Volhard 1988b) and remains an iconic and widely studied developmental regulator. BCD is distributed in an exponentially decaying concentration gradient along the anteroposterior (A–P) axis of embryos and predominantly regulates the activity of ∼100 genes in distinct spatial expression domains ranging from the anterior tip to the middle of the embryo (Driever and Nusslein-Volhard 1988a, 1989; Struhl et al. 1989; Driever et al. 1990).

The ability of BCD and other morphogens to activate different target genes at locations along concentration gradients is classically thought to arise from variations in the number and strength of cognate DNA-binding sites within different enhancers (Burz et al. 1998; Lebrecht et al. 2005; Xu et al. 2015), with sharp expression domain boundaries then set through cooperative binding (Ephrussi and St Johnston 2004; Lebrecht et al. 2005). Under this model, enhancers with lower-affinity binding sites would be activated only at high concentrations, while enhancers with higher-affinity sites would also be activated at lower concentrations. This explains how particular enhancers differentially interpret the same gradient to establish spatial domains of gene expression. In recent years this classical model of concentration-dependent activation has been challenged through experiments on mutant embryos with flattened BCD distributions, which reveal that segment order and polarity can be maintained even without a concentration gradient (Ochoa-Espinosa et al. 2009). It has been suggested that instead of a pure concentration dependence, the activation of BCD target genes and the resulting sharp expression domain boundaries are tightly regulated by spatially opposing gradients of repressors (Chen et al. 2012) and the combinatorial actions of other TFs (Combs and Eisen 2017).

The recent discovery of the ubiquitous factor Zelda (ZLD) and its role in the regulation of chromatin accessibility (Liang et al. 2008; Harrison et al. 2011; Foo et al. 2014; Li et al. 2014; Xu et al. 2014; Schulz et al. 2015; Sun et al. 2015; Blythe and Wieschaus 2016) and in modulating the timing and strength of BCD-controlled (Xu et al. 2014; Hannon et al. 2017) and Dorsal-controlled (Foo et al. 2014) enhancer activation in a concentration-dependent fashion has further strengthened the hypothesis that the interpretation of the BCD and other morphogen gradients is more complex than previously thought (Lucchetta et al. 2005).

Extant models cannot, for example, address how BCD is sufficient for activating its targets such as Knirps (Rivera-Pomar et al. 1995) and Hairy (La Rosee et al. 1997) in the posterior of the embryo, where BCD nuclear concentrations are <2 nM (Morrison et al. 2012) in the short interphase times (5–10 min) of the early nuclear cycles. Since the question of how BCD molecules can find their targets in these short times requires dynamic measurements, genomic assays and biochemical approaches that provide static snapshots have proven inadequate to resolve outstanding mechanistic questions about morphogen activity.

In this study, we address gaps in our understanding of morphogen activity by performing direct measurements of BCD–DNA interactions in vivo by single-molecule imaging. Single-molecule imaging in living cells has been increasingly used in recent years to measure the dynamics of TF–DNA interactions (Liu et al. 2015). However, the techniques commonly used are not suitable for whole embryos and thick tissues. Total internal reflection (TIRF) and highly inclined illumination (Hi-Lo) (Tokunaga et al. 2008), which have enabled single-molecule imaging in monolayer cell cultures, use wide-field excitation geometries and restrict the illumination volume to a small distance above the microscope coverslip in order to limit the excitation of out of focus fluorophores. This confinement of the illumination volume is necessary to achieve the signal to background ratios (SBRs) required for single-molecule detection. Consequentially, if the thickness of the illumination volume is extended to image further away from the coverslip, the SBR degrades as increasingly out of focus fluorophore emissions raise the background level and reduce contrast. This degradation is further exacerbated when imaging highly autofluorescent samples such as embryos or thick tissues.

Lattice light-sheet microscopy (LLSM) was developed recently to overcome these technical barriers (Chen et al. 2014a). The principle of LLSM is to create an excitation light sheet that matches the depth of field of the detection objective such that only fluorophores that are in focus are excited (Chen et al. 2014a). As in all light-sheet microscopes, in LLSM, the excitation and detection objectives are independent and oriented orthogonally to each other. However, unlike conventional light-sheet modalities that use Gaussian beam illumination, in LLSM, an array of Bessel beams is used. Light sheets that are generated with Gaussian beams are generally useful for achieving cellular-level resolution over large fields of views, but a severe tradeoff exists between the thickness of the sheet and field of view (Planchon et al. 2011), and thus they are not suitable for imaging with subcellular resolution. On the other hand, Bessel beams that are optically nondiffracting allow for the generation of light sheets with submicrometer thickness while maintaining a suitable field of view (Planchon et al. 2011). In LLSM, the spacing and phase of Bessel beams in an array are controlled such that their side lobes destructively interfere in order to achieve maximal axial confinement of the light sheet while also minimizing phototoxicity and bleaching by spreading the excitation energy across the array of beams (Chen et al. 2014a). Unlike in wide-field excitation geometries, the thickness of the excitation volume in LLSM is independent of the distance from the coverslip that is being probed.

Here we applied LLSM to developing *D. melanogaster* embryos in order to characterize for the first time the single-molecule DNA-binding kinetics of BCD along its concentration gradient. We found that BCD binds to chromatin in a highly transient manner, with specific binding events lasting on the order of a second, in all portions of the embryo. Examination of the spatial distribution of BCD-binding events reveals spatiotemporal hubs of high local BCD concentration that increase its local DNA binding on rate and facilitate specific binding even with such a high off rate. This effect is most dramatic in posterior nuclei, where, given that there is minimal BCD, we were surprised to observe a significant number of binding events. Through genome-wide analysis of BCD–DNA binding on dissected posterior segments of embryos, we show that the binding that we observed via single-molecule imaging in posterior nuclei occurs at specific regulatory regions, a result that cannot be explained by classical models of BCD activity.

We further found that the regions that are enriched for BCD in the posterior segments are highly correlated with binding of the maternal factor ZLD, which has been shown previously to affect the regulation of BCD targets, especially at lower concentrations. Through single-molecule imaging of BCD in ZLD-null embryos, we show that ZLD is required for the formation of BCD hubs in the posterior embryo. Together, these data advocate for a model in which ZLD mediates the formation of hubs of high local BCD concentration that facilitate BCD binding and enable the activation of BCD-dependent targets at all positions along the A–P axis of the embryo.

## Results

### Single-molecule imaging in living *Drosophila* embryos using LLSM

**Figure 1.**
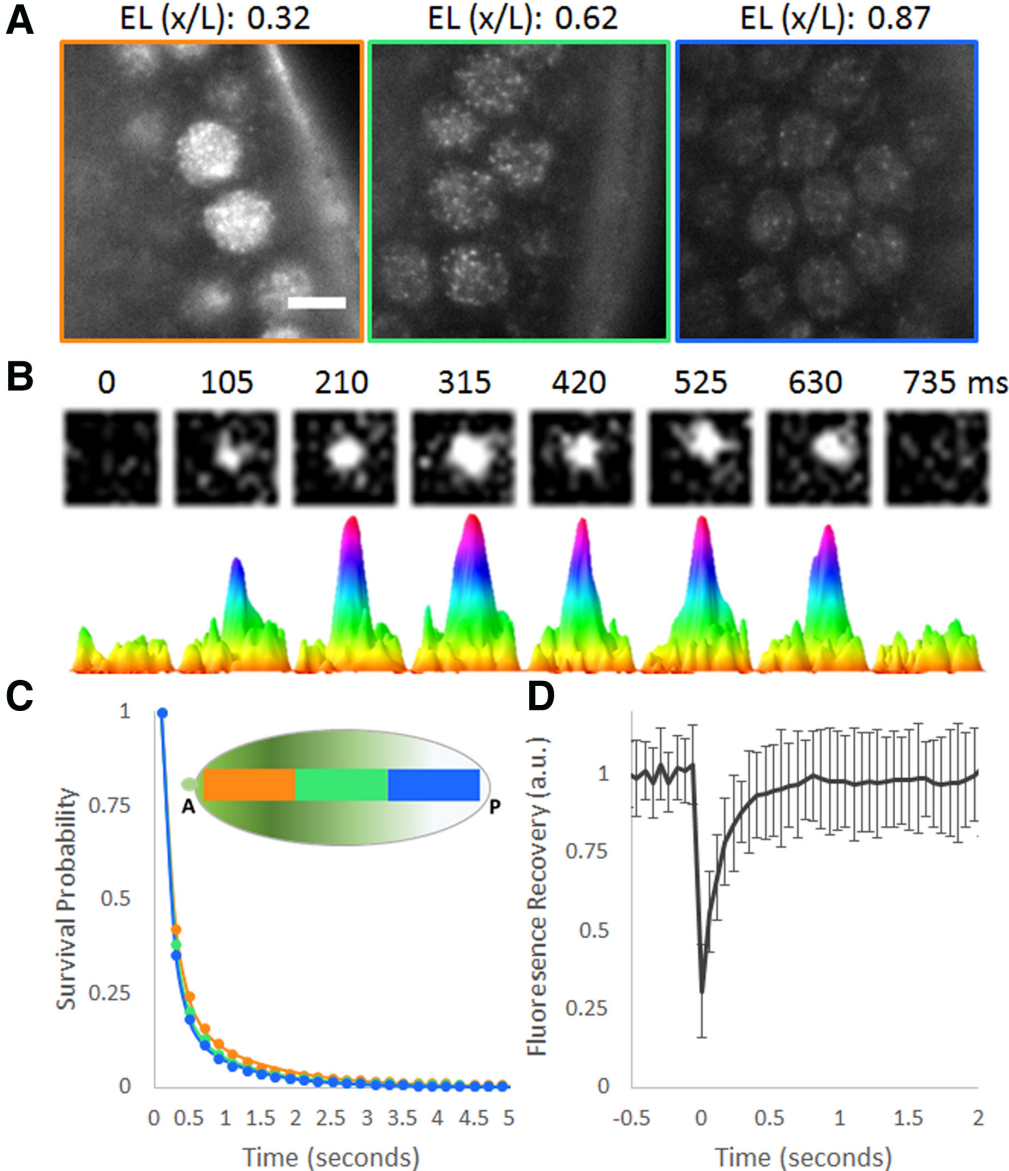
Single-molecule kinetics of BCD in living *Drosophila* embryos. (*A*) Raw images of BCD-eGFP molecules in a living *Drosophila* embryo acquired with a 100-msec exposure time. Bar, 5 µm. Positions along the A–P axis are shown as a fraction of the embryonic length [EL (x/L)]. (*B*) Example of a single-molecule-binding event. The *top* row shows raw images from a 1.2 × 1.2-µm area, and the *bottom* row shows corresponding surface plot representations to illustrate the signal to noise. (*C*) Uncorrected survival probability curves for BCD binding (markers) in the anterior (34 nuclei), middle (70 nuclei), and posterior (83 nuclei) segments of the embryo and corresponding fits to a two-exponent model (solid lines) show no significant differences. (*D*) Fluorescence recovery after photobleaching (FRAP) curve for BCD shows a recovery time on the order of hundreds of milliseconds, and error bars show standard deviation over 21 nuclei.

We constructed a lattice light-sheet microscope (Supplemental Fig. S1; Chen et al. 2014a) and adapted it to image BCD-eGFP in living *Drosophila* embryos over a large field of view with high temporal resolution (Fig. 1A,B; Supplemental Fig. S2; Supplemental Movies 1–3). We used a *yw*; *his2av-mrfp1; bcdE1, egfp-bcd* fly line in which only the labeled BCD is expressed to indicate proper functionality and expression levels (Gregor et al. 2007) and ensure that all molecules that we observed were functionally relevant. The single-molecule nature of the data is reflected in the distribution of intensities (Supplemental Movie 2; Supplemental Fig. S2A,B) and discrete characteristics (Supplemental Movie 2; Supplemental Fig. S2C,D) of the observed binding events.

BCD nuclear concentrations, as measured by two-photon microscopy, on the same fly line that we used in this work, range from ∼50 nM at the anterior-most positions down to <2 nM (Morrison et al. 2012) in the posterior. This translates to a range on the order of 10^4^–10^2^ BCD molecules per nucleus. To gain some preliminary insight into what to expect when imaging with the LLSM, we assumed an isotropic distribution of molecules and a 400-nm-thick excitation sheet and estimated a range on the order of 10^3^–10^1^ BCD molecules per imaging plane in a single nucleus. This simple calculation provides an intuitive feeling of why it is possible to perform single-molecule imaging using BCD-eGFP. This range of concentrations is reflected in the data shown in Supplemental Movie S1, where unambiguous single-molecule detections can be seen in the middle and posterior positions from the start, whereas in the anterior positions, they can be detected only when a sufficient amount of bleaching has occurred. This natural concentration range allows us to perform single-molecule tracking at all positions in the embryo without using sparse labeling strategies or photoswitchable fluorophores.

### BCD binds chromatin in a highly transient manner across the concentration gradient

At high concentrations of BCD in the anterior, the on rate—and thus time average occupancy—is high at both low-affinity and high-affinity binding sites. Under the classical model, the strength of binding sites within specific enhancers is set such that the time average occupancy varies depending on the affinity of binding sites and position along the concentration gradient. Thus, at vanishingly low concentrations in the posterior embryo, the time average occupancy at all enhancers is expected to be low, since even the highest-affinity sites would not be occupied frequently enough to drive expression. To test this model at the single-molecule level, we therefore first performed single-molecule imaging and tracking at long (100-msec) exposure times, effectively blurring out the fast-moving (unbound) population (Supplemental Movies 1–2; Supplemental Fig. S2; Chen et al. 2014b) to estimate the residence times (RTs) of BCD binding in nuclei at all positions along the A–P axis.

Previous single-molecule studies of TFs have consistently found two populations in the survival probability distributions: a short-lived population with RTs on the order of hundreds of milliseconds and a longer-lived population with RTs on the order of tens of seconds to minutes (Chen et al. 2014b; Normanno et al. 2015; Hansen et al. 2017). These two populations have often been shown to be the nonspecific and specific binding populations, respectively. The survival probability distributions of BCD similarly are fit better with a two-exponent model than a single-exponent model, indicating the presence of two subpopulations (Supplemental Fig. S3). Fits to the survival probability distributions of BCD-binding events (Fig. 1C; Supplemental Table 1) in the anterior, middle, and posterior thirds of the embryo identified short-lived populations with average RTs (after photobleaching correction) on the order of hundreds of milliseconds and longer-lived populations with average RTs on the order of 1 sec, with no significant dependence on position along the A–P axis for either population (Supplemental Table 1). The validity of our RT estimation of specific binding on the order of 1 sec is supported by additional measurements at 500-msec exposure times (Supplemental Fig. S4; Supplemental Table 1). We note that the values reported here are the genome-wide averages for both the long-lived and short-lived binding populations. It is also likely that there are even shorter-lived BCD–DNA interactions that we cannot access due to the practical trade-off between temporal resolution and signal to noise ratio in single-molecule imaging.

To further validate our observation that the RT of the long-lived population of BCD is highly transient compared with the 10–60 sec typically observed for other sequence-specific DNA-binding TFs using single-molecule tracking (Chen et al. 2014b; Hansen et al. 2017), we performed fluorescence recovery after photobleaching (FRAP) experiments on BCD in the embryo (Fig. 1D), which revealed halftimes of BCD recovery on the order of hundreds of milliseconds (Supplemental Fig. S5). The rapid dynamics of BCD indicated by the FRAP data are consistent with previous measurements by others using fluorescence correlation spectroscopy (FCS) (Porcher et al. 2010). Although the FCS measurements on nuclear BCD dynamics (Porcher et al. 2010) were previously analyzed only to estimate the diffusion coefficients of fast-moving and slow-moving populations, when these data are reanalyzed using a “stick and diffuse” model (Yeung et al. 2007), which takes into account binding events between diffusion, the RT for a short-lived binding population of 122 msec is found, consistent with our measurements (Fradin 2017). These results confirm the transient nature of BCD binding; however, due to the limitations on the dynamic range of FCS measurements and the kinetic modeling used, the less prevalent longer-lived binding population that we quantified with single-molecule imaging cannot be detected. The dominance of the short-lived interactions (Fig. 1C) highlights the preponderance of low-affinity BCD-binding sites in the *D. melanogaster* genome and the resulting large number of nonspecific interactions, as suggested previously by genomic studies (Ochoa-Espinosa et al. 2005; Li et al. 2008). The observation of a significant number of binding events in posterior nuclei is surprising, as we expected the majority of the few BCD molecules left to be diffusing and binding too infrequently to specific targets to be detected.

### Spatiotemporal hubs of BCD binding enrich local concentrations in the posterior embryo

**Figure 2.**
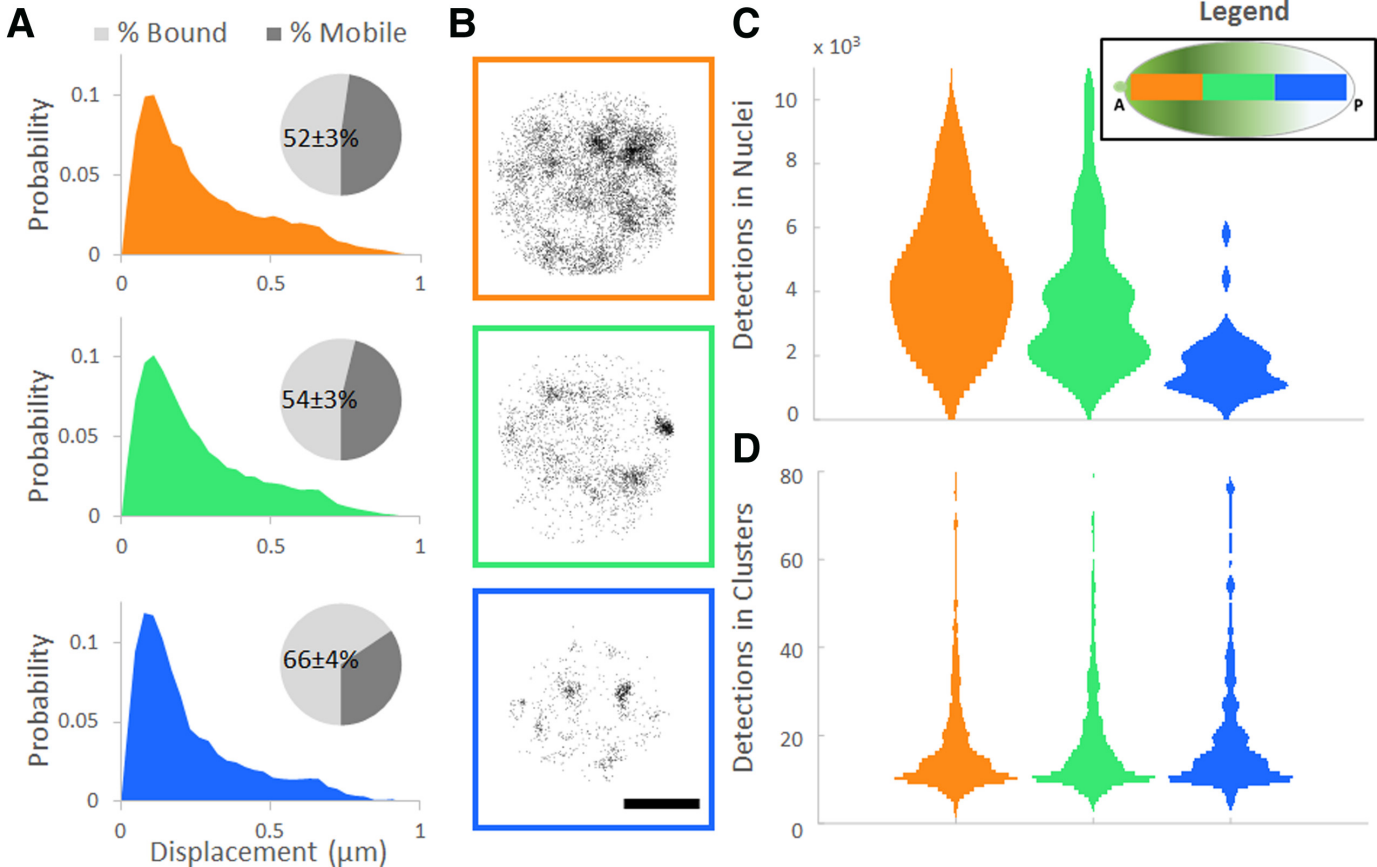
Local modulation of BCD concentration. (*A*) Normalized probability distributions of measured displacements in the anterior (30 nuclei), middle (67 nuclei), and posterior (66 nuclei) positions of the embryos; pie charts show the estimated mobile and bound fractions from fits to a two-population distribution, with the bound population percentage labeled with the standard error of the fit parameter. (*B*) Examples of the spatial distribution of all detections in nuclei along the A–P axis. Bar, 2.5 µm. (*C*) Distribution of the number of detections in all nuclei. (*D*) Distributions of the number of detections within all clusters.

The observation of significant binding events in the posterior embryo, where BCD has been reported to be at vanishingly low (<2 nM posterior vs. ∼50 nM anterior) concentrations (Morrison et al. 2012), motivated us to next measure the fraction of the BCD population that is diffusing versus bound along the concentration gradient. Since longer exposure times allow detection of only molecules bound for at least the span of the exposure and do not provide any data on the mobile population that is blurred into the background, we performed single-molecule tracking measurements at a decreased exposure time of 10 msec. The signal to noise ratios at these lower exposure times are adversely affected as expected, limiting the type of analysis that can be performed on these data (Supplemental Movie 3). However, despite this reduced contrast, we were able to perform single-particle tracking and, through analyses of displacement distributions (Supplemental Fig. S6), estimated the fraction of BCD that is bound along the A–P axis (Fig. 2A). Surprisingly, a greater fraction of the BCD population is bound in more posterior positions of the embryo, where BCD is present at the lowest concentrations.

This counterintuitive result, which suggests that the on rate of BCD–DNA binding is decoupled from its nuclear concentration, motivated us to re-examine the 100-msec exposure time data set. One way to resolve this discrepancy would be for BCD to be restricted to a small volume within the nucleus, increasing its effective local concentration. Indeed, when we analyzed the spatial distribution of BCD-binding events in the 100-msec data set, we saw a distinct spatiotemporal clustering (Supplemental Movie S4) of binding events that became more pronounced toward posterior positions (Fig. 2B; Supplemental Fig. S7), with a greater fraction of binding events occurring within clusters (Supplemental Fig. S8). Remarkably, although the number of binding events per nucleus followed the trend dictated by the global concentration gradient across the embryo (Fig. 2C), the distribution of BCD molecules detected per cluster was maintained even in the posterior (Fig. 2D).

Together, these data suggest that BCD forms hubs with high local concentration that lead to high time-averaged occupancy at specific sites in nuclei across the A–P axis. More hubs are formed at higher concentrations, but the characteristics of hubs are independent of position along the BCD concentration gradient. The surprising observation that BCD binding is concentrated in hubs led us to next ask whether there is a mechanisms to enrich functional BCD target sites in these regions. To do this, we concentrated on posterior positions, where the majority of binding events is occurring within the hubs.

### BCD binds specific regulatory regions in the posterior embryo in a ZLD-dependent manner

**Figure 3.**
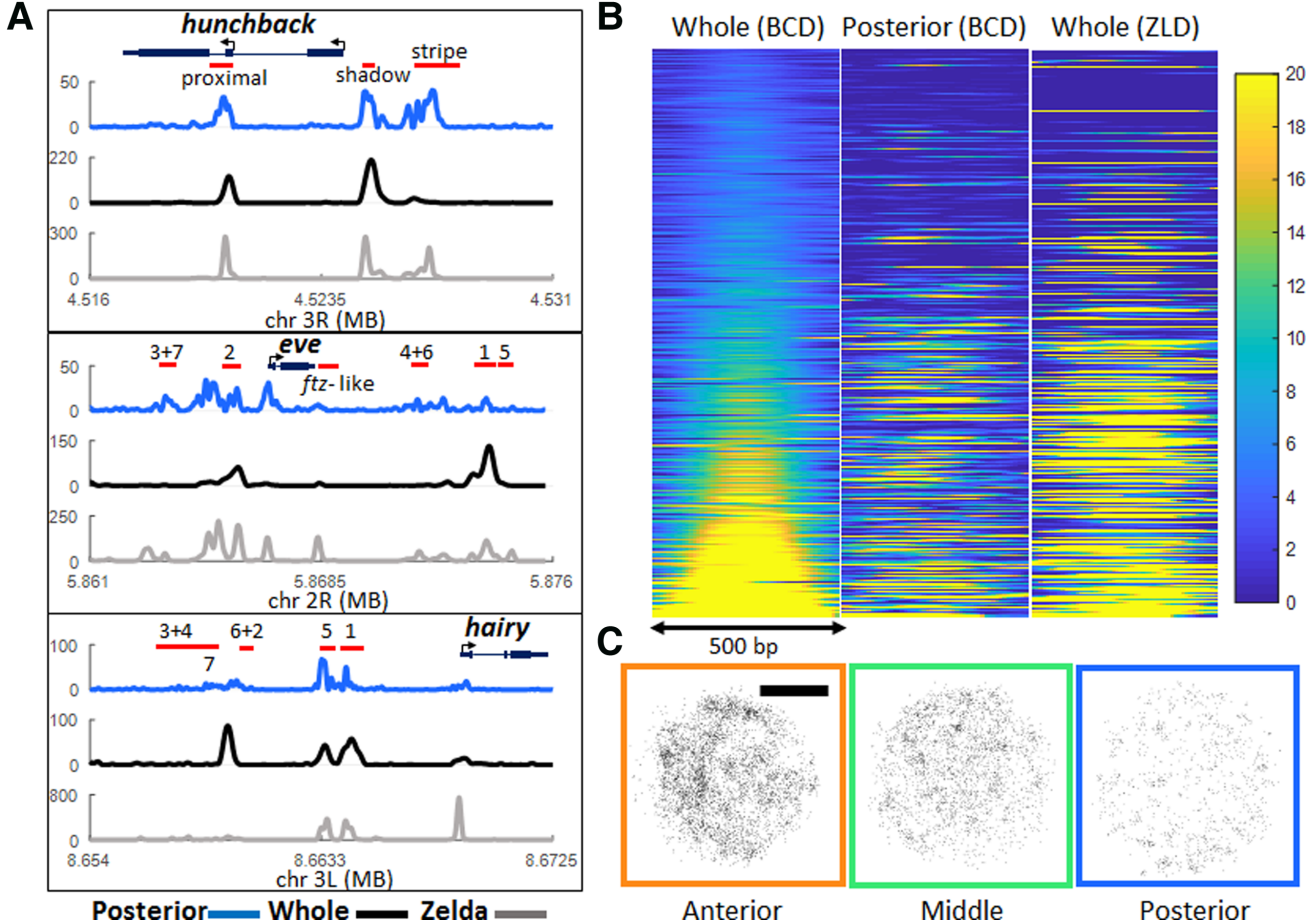
ZLD mediated BCD binding in the posterior embryo. (*A*) Posterior third (blue) and whole embryo (black) BCD and whole embryo ZLD (gray). ChIP-seq signal-normalized reads at the *hunchback*, *eve*, and *hairy* gene loci. Red bars show known enhancers as annotated in the RedFly database; for eve and hairy, they are numbered according to the stripes that they are thought to regulate. (*B*) Heat map representation of normalized BCD ChIP-seq reads (first two panels) and ZLD ChIP-seq reads (third panel) in a 500-base-pair window centered on BCD peaks called in the whole-embryo data and sorted according to increasing signal of the whole-embryo data; a total of 2145 peaks is shown, and colors indicate enrichment over the background (blue), with all plots displayed on the same scale. (*C*) Examples of the spatial distribution of all detected bound molecules in nuclei along the A–P axis in ZLD embryos. Bar, 2.5 µm. A loss of clustering is apparent compared with the distributions shown in Figure 2B.

To test whether BCD is binding with specificity in the posterior embryo, we analyzed its binding profiles in a spatially segregated manner (Combs and Eisen 2013) by comparing ChIP-seq (chromatin immunoprecipitation [ChIP] combined with high-throughput sequencing) profiles derived from individually dissected posterior thirds of embryos with previously published data from whole embryos (Bradley et al. 2010). Our analysis reveals that BCD indeed binds to known targets in the posterior but with increased relative enrichment at specific enhancer elements (Fig. 3A). For example, in the *hunchback* locus, binding at the posterior stripe enhancer (Perry et al. 2012) is highly enriched over the background in nuclei from the dissected posterior third relative to the whole embryo. Intriguingly, genomic regions that exhibit a relative increase in BCD occupancy in the posterior are correlated with an enrichment of ZLD binding (Fig. 3A; Supplemental Fig. S9), a ubiquitous activator often described as a pioneer factor active during early embryonic development (Liang et al. 2008; Harrison et al. 2011; Foo et al. 2014; Xu et al. 2014).

Remarkably, enrichment of ZLD is more predictive of BCD binding in the posterior than previously determined positions of enhancer activity for the loci shown in Figure 3A. Analysis of the correlation between BCD and ZLD enrichment at the *cis*-regulatory modules of 12 gene loci and at ZLD and BCD peaks genome-wide reveal that binding of BCD in posterior nuclei is highly correlated with ZLD cobinding (Fig. 3A; Supplemental Figs. S9, S10). The difference in the binding profiles of the posterior third segments compared with whole embryos emphasizes the need to perform genomic analysis in a spatially resolved manner across the A–P axis. While we provide a proof of principle on how to perform these measurements through manual dissection, improved methods are required in order to improve the throughput of these experiments.

### Formation of BCD hubs in the posterior embryo is dependent on ZLD

The posterior third genomic data and the published evidence for ZLD's role in the regulation of chromatin accessibility (Liang et al. 2008; Harrison et al. 2011; Foo et al. 2014; Li et al. 2014; Xu et al. 2014; Schulz et al. 2015; Sun et al. 2015) and its suggested role in the modulation of TF binding at low concentrations (Xu et al. 2014; Schulz et al. 2015) naturally led us to hypothesize that the observed clustering of BCD-binding events may be mediated in part by ZLD. We thus generated *zelda*-null embryos with BCD-eGFP and measured BCD binding at 100-msec exposure times. We found an abolishment of BCD hubs in the posterior embryo and a small decrease in RTs (Fig. 3C; Supplemental Table S1). Due to this loss of clustering, the same analysis that was performed in the wild-type case (Fig. 2C) could not be performed in the ZLD mutants. We thus calculated the pair correlation function for the spatial distribution of binding events in both the wild type and ZLD mutants (Cisse et al. 2013). This analysis allowed us to infer whether binding events are spatially randomly distributed or clustered (Supplemental Fig. S11). Both the magnitude and correlation length indicate a diminishment of clustering in the posterior nuclei of the ZLD mutants. We also note that due to the lower labeling density of BCD in the ZLD-null embryos, the presumably ZLD-independent clustering in the anterior embryo now becomes more apparent (Supplemental Fig. S11). The loss of clustering in the ZLD mutants also confirms that the clustering that we originally observed is not due to aggregation of eGFP but simply nonhomogenous distribution of BCD within nuclei or other artifactual reasons. To provide further validation that the observed clustering is not occurring by chance, we calculated the pair correlation function for randomly distributed points in a disc approximately the diameter of the nuclei for comparison (Supplemental Fig. S11).

**Figure 4.**
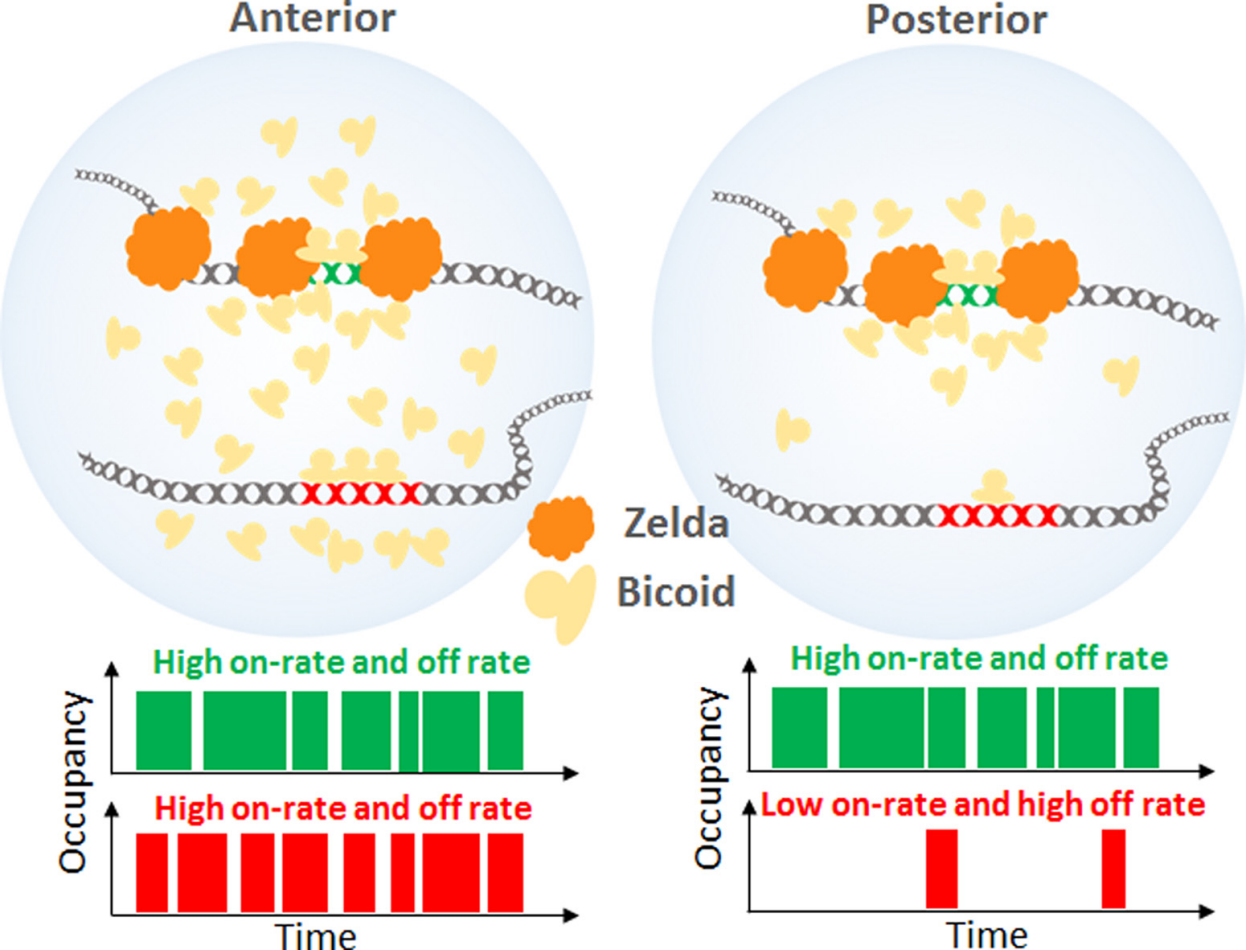
Model of ZLD-dependent modulation of the BCD on rate at specific loci in the posterior embryo. At high concentrations in the anterior of the embryo, all target sites are highly occupied. At low concentrations, loci with ZLD occupancy have an increased time-averaged occupancy through the formation of spatiotemporal hubs that enrich local concentrations and increase the on rate.

Although the exact mechanism by which ZLD mediates BCD hub formation and binding remains unclear, we speculate that a combination of protein–protein interactions facilitated by intrinsically disordered low-complexity domains (Hamm et al. 2015) of ZLD and its reported role in promoting chromatin accessibility (Foo et al. 2014; Li et al. 2014; Schulz et al. 2015; Sun et al. 2015) may contribute to BCD clustering (Fig. 4).

## Discussion

Our initial observation of the low-affinity nature of BCD binding to chromatin is partially congruent with the classical view of BCD as a concentration-dependent morphogen. High BCD concentrations in the anterior embryo lead to high on rates along with high chromatin occupancy, with the high off rate enabling frequent sampling of both specific and nonspecific sites (Fig. 4). As the BCD concentration decreases posteriorly along the gradient, there should be progressively lower on rates and reduced binding to specific sites regardless of the roles of opposing repressor gradients. However, if this is all there is to BCD binding, then this would consign BCD to have no binding or function in more posterior positions, contradicting a wealth of evidence pointing to a specific role for BCD in the regulation of posterior gene expression (Rivera-Pomar et al. 1995; Small et al. 1996; La Rosee et al. 1997; Burz et al. 1998; Ochoa-Espinosa et al. 2009; Chen et al. 2012).

Our demonstration that BCD overcomes the combination of low concentrations and high off rate by forming transient hubs highlights the power of dynamic, single-molecule studies on living embryos. Although most vivid in posterior nuclei, this phenomenon is likely to play a significant role in regulating the large number of important known BCD targets in the middle of the embryo. Unlike BCD targets in the most anterior region, a large number of BCD targets in the middle of the embryo are dependent on ZLD, and our in vivo imaging data provide an elegant model for how this is accomplished, in which ZLD binds to BCD targets, triggers the clustering of BCD (likely via low-complexity region-mediated interactions among ZLD molecules and between ZLD and BCD), thereby increasing the effective concentration of BCD at its targets. We speculate that this protein–protein interaction-mediated clustering acts in tandem with ZLD's known role in promoting chromatin accessibility to facilitate binding to low-affinity enhancers.

The formation of such clusters or hubs has been reported for other TFs (Chen et al. 2014b; Liu et al. 2014; Crocker et al. 2017) and for RNA polymerase II (Cisse et al. 2013), indicating that such spatial organization of the nucleus may be a general mechanism to catalyze important regulatory interactions. During embryonic development, it is likely that clustering of TFs mediated by cofactors has evolved to allow exquisite spatial and segmental modulation during development through enabling interactions with low-affinity enhancers (Crocker et al. 2016).

## Materials and methods

### Fly husbandry

All fly cages were prepared by combining males and females of the desired strains in a plastic cage left for at least 3 d at room temperature in light prior to imaging. The lids on the cages were filled with agar dissolved in apple juice (2.4% [g/w] Bacto agar, 25% apple juice, 75% distilled water, 0.001% mold inhibitor from solution of 0.1g/mL [Carolina, 87-6165]). A paste of dry yeast was smeared on the lids to induce egg laying. Lids were exchanged once each day.

The fly strain used for all wild-type BCD imaging experiments was *yw*; *his2av-mrfp1; BcdE1, egfp-bcd*. This fly line results in embryos in which only the labeled BCD is expressed, indicating proper functionality and expression levels (Gregor et al. 2007). For the *zld*^−^ experiments, *bcd-egfp* heterozygous virgins with *zld*^−^ germline cells (maternal germline clones prepared as in Liang et al. (2008) were crossed to *yw* males, and progeny were used for imaging 2–3 h after laying. The heterozygosity results in only half of the BCD labeled in the *zld*^−^ embryos. For photobleaching controls, the line used was *yw, his2av-egfp*; +/+ (Bloomington no. 24163).

### Live embryo collection for imaging

For embryo collection, lids on fly cages were exchanged 1 h prior to imaging. After 1 h, embryos were collected from the lids using an inoculation loop. A 5-mm-diameter glass coverslip (Warner Instruments, no. 64-0700) was prepared by immersion in a small amount of glue (prepared by dissolving adhesive from about one-fifth of a roll of double-sided Scotch tape overnight in heptane) and left to dry for 5–10 min while collecting embryos. Collection was performed on a dissection scope with transillumination. Embryos were bathed in Halocarbon oil 27 (Sigma) for staging and then selected between developmental stages 1 and 4. Selected embryos were placed on a small square of paper towel and then dechorionated in 100% bleach for 1 min. Bleach was wicked off with a Kimwipe after 1 min, and then the square was washed with a small amount of distilled water. Excess water was wicked off the square, and the square was dipped in a small water bath. Unpunctured embryos that floated to the top of the bath were selected for imaging and placed on a small paper towel square to slightly dry. To prevent excess desiccation, embryos were immediately placed on the glass coverslip in rows and then immersed in a drop of phosphate-buffered saline (PBS).

### LLSM

Imaging was performed using a home-built lattice light-sheet microscope (Supplemental Fig. S1) following the design described by Chen et al. (2014a) and detailed blueprints provided by the Betzig group at the Howard Hughes Medical Institute Janelia Research Campus. To perform the single-molecule experiments, we added a detection module containing two EMCCDs (Andor iXon Ultra) for dual-color imaging. The EMCCDs provided a significant improvement in signal to noise over the sCMOS (Hamamatsu Orca Flash version 2.0) used in the original system and made it possible to use lower excitation powers while maintaining single-molecule sensitivity. In brief, the output beam from each laser was expanded and collimated independently to a size of 2.5 mm. The expanded beams for each laser were combined and input into an acoustic optical tunable filter (AOTF) to allow for rapid switching between excitation wavelengths and adjustment of power (Supplemental Fig. S1A). A pair of cylindrical lenses was then used to elongate and collimate the output Gaussian beam to illuminate a stripe on a spatial light modulator (SLM). The SLM was used to generate a coherent pattern of an array of 30 Bessel beams spaced such that they coherently interfered to create a two-dimensional (2D) optical lattice pattern with a maximum numerical aperture (NA) of 0.6 and minimum NA of 0.505. A 500-mm lens was used to project the Fourier transform of the SLM plane onto an annular mask conjugate to the back pupil plane (BPP) of the excitation objective to spatially filter the pattern (Supplemental Fig. S1B). The BPP was then projected first onto a galvo scanning mirror for *z*-scanning and then onto a second galvo scanning mirror for x-dithering. The x-galvo scanning plane was projected onto the BPP of the excitation objective (Supplemental Fig. S1C). The excitation objective focused the lattice pattern onto the sample, exciting any fluorophores within the axial range (∼400 nm) of the sheet. The emitted fluorescence was collected by the detection objective, which was oriented orthogonally to the excitation objective and projected onto an intermediate image plane by a 500-mm tube lens (Supplemental Fig. S1D). An 80- and 200-mm lens pair was then used to demagnify the image further to provide a 100-nm sampling per pixel on each of the EMCCD sensors. A dichroic mirror (Semrock, FF560-FDi01) was placed between the last lens pair to allow for dual-color imaging in red and green with maximal spectral separation. An emission filter was placed in the path of each camera to both reject the excitation wavelengths and select the wavelength range of interest (Semrock, FF03-525/50 for eGFP and FF01-593/46 for RFP) (Supplemental Fig. S1E). During each camera exposure, the x-galvo mirror was dithered twice over a 5.1-µm range in 100-nm steps to provide uniform illumination.

The prepared coverslip, with embryos arranged in rows as described above, was then loaded into the sample holder and secured onto the positioning stages of the lattice light-sheet microscope. The sample chamber was filled with PBS for imaging and kept at room temperature. The slide was then scanned to find an embryo of suitable age (between nuclear cycles 10 and 11), and the positions of the anterior and posterior extremes of the embryo were then marked. For each data set acquired, the stage position was recorded to determine the position as a fraction of the embryonic length (EL) as the distance of the position from the anterior pole divided by the total length of the embryo.

For the RT measurements on BCD-eGFP, a 488-nm excitation laser was used with a power of 2.9 mW measured at the back pupil plane of the excitation objective. Images were acquired with 100-msec exposure times and an EM gain setting of 300. At each location, at least 1000 frames were acquired, resulting in a total time of 105 sec with a frame rate of 105 msec. Prior to and after acquiring the BCD-eGFP data, an image was taken in His2-AVmRFP using a 561-nm excitation laser at an excitation power of ∼0.17 mW at the back pupil plane to determine the nuclear cycle phase; data not acquired during interphase were discarded upon examination of these images. For RT measurements at 500-msec exposure times, the 488-nm excitation laser power at the back pupil plane was reduced to 0.5 mW; all other settings were the same as above. For the displacement distribution measurements, the exposure time was set to 10 msec, resulting in a frame rate of 15 msec, and the excitation power was increased to 8.28 mW for the 488-nm laser line. All other settings were the same as described above. Viability of the embryos was determined by allowing them to develop until gastrulation after imaging. For the *zelda* embryos, lethality was confirmed after imaging.

### Curation of data for analysis

For all data sets, the following procedure was followed: First, for each movie, the corresponding before and after histone images were checked for any evidence of chromatin condensation to ensure that analysis was performed only in interphase nuclei. Data from mid to late nuclear cycle 14, where the nuclei exhibit an elongated shape, were also excluded. A metadata file was then created for each movie file containing the position as a fraction of the EL (0 for anterior and 1 for posterior), the nuclear cycle (determined by counting the number of mitoses before the 14th cycle). Visual examination of the data set was used to determine whether there was any motion of the nuclei during the acquisition period. Movies that contained any detectable motion were discarded or cropped to include only the time interval in which there was no motion. A rectangular region of interest was then drawn around each nucleus that was then used to crop areas around individual nuclei. The boundary of each nucleus was then marked using a hand-drawn polygon. A masked movie was then created for each nucleus in which regions outside the nucleus were set to 0 grayscale values so that all of the analyses described below were performed only on molecules within nuclear regions.

### Single-molecule localization and tracking using dynamic multiple-target tracing (MTT)

Localization and tracking of single molecules were conducted using a Matlab implementation of the MTT algorithm (Serge et al. 2008). In brief, the algorithm first performed a bidimensional Gaussian fitting to localize particles constrained by a log-likelihood ratio test subject to a localization error threshold. Deflation looping was performed to detect molecules that were partially overlapping. The parameters of the localization and tracking algorithms were empirically determined through iterative examination of the results. For all data sets, the following settings were used: For localization, the maximum number of deflation loops was set to 10, and localization error was set to 10^−6^. For tracking, the maximum expected diffusion coefficient was set to 5 µm^2^/sec, the maximum number of competitors was set to 1, and the maximum off/blinking frames was set to 1.

### RT analysis

The RTs were estimated from the 100-msec data using the results from the single-particle tracking using the MTT algorithm. The data were pooled into bins corresponding to the position along the A–P axis of the embryo in one-third fractions of the EL (0–1 anterior to posterior) with the following number of nuclei and single-molecule trajectories per position bin: anterior: 34 nuclei, 17,735 trajectories; middle: 70 nuclei, 40,092 trajectories; and posterior: 83 nuclei, 20,823 trajectories.

In the ZLD embryos, we measured the following number of nuclei and single-molecule trajectories per position bin: anterior: 23 nuclei, 11,415 trajectories; middle: 31 nuclei, 7572 trajectories; and posterior: 31 nuclei, 3606 trajectories.

The survival probability distribution was then calculated as 1 − the cumulative distribution function of the trajectory lengths and was fit to both single- and double-exponential models (Mazza et al. 2012). The double-exponential model fit the data better in all cases (Supplemental Figs. S3, S4). The model used to fit the data and calculate the time constants and fraction of the population was

$$\text{surival probability}(t)=A[F_{a}e^{-k_{s}t}+(1-F_{a})e^{-k_{ns}t}],$$

where *k*_*s*_ and *k*_*n*__s_ are the low (specific) and high (nonspecific) off rates, respectively. The total pooled data sets of 78,650 trajectories from the MTT results from 187 nuclei were also fit in the same manner.

To correct for photobleaching, we used *His2Av-eGFP* to estimate the bleaching constant (0.00426 sec^−1^) and correct the off rate as *k*_s,corrected_ = *k*_s_−*k*_bleach_.(Supplemental Table S1). We note that the bleaching correction had a minimal effect on our estimated off rates. The fact that we were not limited by bleaching due to the transient nature of BCD binding was further validated through even longer, 500-msec exposure time measurements on 17 nuclei, which provided an estimate for the specific and nonspecific off rates that did not differ significantly from those measured at 100 msec (Supplemental Fig. S4; Supplemental Table S1). The results of the fits to all data are shown in Supplemental Table S1.

### FRAP

Experiments were performed on a Zeiss LSM 800 laser scanning confocal system (coupled to a Zeiss Axio Observer Z1) using a plan-achromat 63×/1.4 NA oil immersion objective, a GaAsP-PMT detector, and a 488-nm laser. A circular bleach region with a diameter of 1.5 µm was used, the bleach location was selected manually in each nucleus at approximately the center, and a total bleach time of 78.1 msec was used. Data were acquired for at least 1 sec prior to bleaching and at least 6 sec after bleaching, with a time interval of 0.430 msec. Experiments were performed using live embryos from the same fly line as for lattice light-sheet imaging and were collected and prepared in the same manner as described above with the exception of the mounting procedure. For FRAP, the embryos were mounted between a semipermeable membrane (Biofolie, In Vitro Systems and Services) and a coverslip and then embedded in Halocarbon 27 oil (Sigma). As in the case of the single-molecule measurements, the embryos were staged using the HIS2-AV-MRFP channel, and all data were acquired on embryos in nuclear cycle 13. The data were acquired on 21 nuclei, all within the first 25% of the embryonic length from the anterior pole. FRAP experiments could not be performed at more anterior locations due to the low BCD concentrations and thus signal to noise ratio.

For analysis, the spatial and temporal locations of the bleach region were retrieved from the metadata recorded by the microscope control software and manually verified by inspecting the data. Due to the short duration of the movies and rapid recovery, drift correction was not necessary. Each nucleus was manually segmented from the rest of the image by defining a polygon region of interest. A region of interest the same size as the bleach region was also marked in an area of each image outside of the nuclear region to be used to measure the background or dark intensity. The FRAP curve for each nucleus was then calculated as follows:

First, the mean intensity in the nuclear region, *I*_nuc_(*t*), bleach region, *I*_bleach_(*t*), and background region, *I*_dark_(*t*), was calculated at each frame.

The background intensity was then subtracted from both the mean nuclear and bleach region intensities. To correct for photobleaching from imaging, the bleach region intensity was then divided by the mean nuclear intensity at each time point. The resulting photobleaching-corrected intensity was then normalized to the mean prebleach intensity, calculated as the average intensity in all frames prior to bleaching (*I*_prebleach)_. The final FRAP curve for each nucleus was thus calculated as FRAP(*t*) = {[*I*_bleach_(*t*) − *I*_dark_(*t*)]/[*I*_nuc_(*t*) − *I*_dark_(*t*)]}*I*_prebleach_.

The calculated FRAP curves for each nucleus were then aligned to the bleach frame and averaged to generate the average FRAP curve shown in Figure 1D. The averaged recovery data were then fit to both a single-exponential [1 − A × exp(−*ka* × *t*)] and double-exponential [1 − *A* × exp(−*ka* × *t*) − *B* × exp(−*ka* × *t*)] model (Supplemental Fig. S5A,B) to measure the recovery time constants. There was no significant difference in the quality of fit between the two models. For comparison, the exact same experimental and analysis procedure was followed for His2AV-MRFP1 (Supplemental Fig. S5C) in the same embryos with the exception of using a 561-nm bleach laser; three nuclei were measured with these settings. For the histone measurements, the two-exponent fit was significantly better than the one-exponent, as expected.

### Displacement distribution analysis

Single-molecule trajectories were analyzed as described above. A total of 158 nuclei from four embryos was analyzed. The data from nuclei were binned according to their position along the A–P axis in one-third fractions of the EL (0–1, anterior to posterior) with the following number of nuclei and single-molecule displacements per bin: In the anterior-most (0–0.2) positions, tracking could not be performed reliably due to the high concentrations of BCD at those locations at these high frame rates and presumably a large mobile population: anterior: 30 nuclei, 12,923 trajectories; middle: 67 nuclei, 23,640 trajectories; and posterior: 66 nuclei; 8600 trajectories.

The fraction of the population that is bound versus mobile was estimated using two approaches. First, a cumulative distribution function of the displacements was calculated for each EL bin (Supplemental Fig. S6); displacements corresponding to distances <225 nm were scored as part of the bound population. In the second approach, the probability density functions of the displacements were fit to a two-population model (Supplemental Fig. S6B):

$$P(r)=F_{\mathrm{bound}}\frac{r}{A}e^{\frac{r^{2}}{2A}}+(1-F_{\mathrm{bound}})\frac{r}{B}e^{\frac{r^{2}}{2B}},$$

where *F*_bound_ is the fraction of the population that is considered bound, and *r* is the displacement distance. The two-population model fits the displacement data with *R*^2^ values of 0.97, 0.98, and 0.96 for the anterior, middle, and posterior distributions, respectively, and provides an estimate of the fraction present in the mobile and bound populations. The trend of an increase in the population-bound estimate in the middle and posterior positions relative to the anterior is similar from both approaches. Although the bound population includes nonspecific binding events as well, we were interested in the relative change across the A–P axis.

We note that the diffusion coefficients for the bound and mobile populations can, in principle, be estimated from displacement distributions; however, as explained by Mazza et al. (2012), they cannot be estimated accurately from a fit to a displacement distribution at a single time step and are thus not reported. To accurately measure the diffusion coefficients, more stable and photoswitchable fluorophores are necessary to be able to track single molecules for more time points and at higher temporal resolutions.

### DBSCAN (density-based spatial clustering of applications with noise)-based analyses of clusters

The clustering of BCD-binding events in the 100-msec wild-type embryo data set is readily apparent in nuclei across the A–P axis in the projection of all localizations from the MTT algorithm (Fig. 2B). In order to automatically identify clusters and count the number of detections per cluster versus the whole nucleus (Fig. 2C), a Matlab implementation (Yarpiz Team 2015, http://yarpiz.com/255/ypml110-dbscan-clustering) of the widely used DBSCAN was used with a minimum points setting of 10 points and an ε (maximum radius of neighborhood) setting of 0.8. These settings were empirically determined by iteratively changing parameters and examining the results. A balance had to be struck in the ability to accurately identify clusters in the high-density situations in anterior nuclei and also low-density situations in the posterior. Only data sets with time spans of at least 105 sec were included. Totals of 12, 49, and 48 nuclei and 436, 1168, and 367 clusters were analyzed in this manner for the anterior, middle, and posterior positions, respectively. Examples of clusters identified in nuclei at various positions are shown in Supplemental Figure S7. For comparing distributions, outliers were removed (<5th or >95th percentile) to disregard clusters that were significantly overpartitioned or underpartitioned. In the case of the ZLD^−^ embryos, due to the loss of apparent clustering, DBSCAN was not able to detect clusters, so instead the spatial distribution of points was compared using pair correlation analysis as described below to provide insight into the change beyond visual examination.

### Pair correlation analysis of clustering

The pair correlation function for the spatial distribution of particles computes the probability of finding a particle at the range of distances from other particles. In the case of complete spatial randomness, which can be represented by a Poisson process, the pair correlation function is equal to 1. The analysis is conducted on point lists generated from the MTT algorithm using the first spatial coordinate of each trajectory. Totals of 22, 48, and 42 nuclei were analyzed for the anterior, middle, and posterior positions, respectively, for the wild-type embryos and totals of 23, 31, and 31 nuclei were analyzed for the anterior, middle, and posterior positions, respectively, for the *zld* embryos. A Matlab implementation was used to calculate the correlation function for the spatial distribution in each nucleus. The results were then averaged for each position (anterior, middle, and posterior) embryo for comparison (Supplemental Fig. S11). To generate simulations of randomly distributed points, spatial coordinates of detections were generated randomly (using a Matlab script and random draws from a uniform distribution) to lie within a 5-μm disc, and simulations were performed with 5000, 3000, and 1000 points to correspond to detections at anterior, middle, and posterior positions.

### ChIP-seq

Embryos were collected from a population cage for 90 min and then aged for 2 h in order to enrich for embryos at developmental stage 5. Embryos were then fixed with formaldehyde as described previously (Li et al. 2008) and sorted by morphology for those at early stage 5. The posterior thirds of embryos were sliced off by hand with a scalpel. A pool of the embryo segments from ∼300 embryos was combined with 20 µg of whole *Drosophila pseudoobscura* embryos at stage 5 (to serve as carrier) and homogenized in homogenization buffer containing 15 mM NaCl, 15 mM Tris-HCl (pH 7.5), 60 mM KCl, 1 mM EDTA, and 0.1% Triton X-100, with 1 mM DTT, 0.1 mM PMSF, and protease inhibit cocktail (Roche) added before use. After homogenization, 0.5% NP40 was added, and, following a 5-min incubation, samples were spun down at a low speed. Nuclei in the pellet were then washed once with the homogenization buffer containing 0.2 M NaCl. The low-speed centrifugation was repeated, and the recovered nucleus pellet was then resuspended in nuclear lysis buffer (10 mM TrisCl at pH 7.9, 100 mM NaCl, 1 mM EDTA, 0.5% sarkosyl), 1% SDS, and 1.5% sarkosyl. The chromatin was recovered by spinning the sample at full speed in a microcentrifuge for 1 h at 4°C and was resuspended in a small volume of nuclear lysis buffer. Chromatin was sheared to an average size of 300 base pairs (bp) using a Covaris sonicator (peak power, 140; duty factor, 2; cycle burst, 200; time, 2:20 min). ChIP was performed using 72 ng of chromatin and 1.5 µg of an anti-BCD polyclonal antibody described previously (Li et al. 2008). The BCD antibody was coupled to Dynabead M-280 sheep anti-rabbit magnetic beads, and the immunoprecipitation was conducted with the standard protocol from the manufacturer. DNA libraries for the ChIP samples were prepared using the Rubicon genomics Thru-Plex DNA-seq kit using 16 PCR cycles and sequenced using Illumina High-seq with 2500 rapid-run 100-bp single-end reads. The sequencing reads were aligned to a combined *D. pseudoobscura* (Flybase release 1.0) and *D. melanogaster* (Flybase release 5) genome using Bowtie with options set at -5 5 -3 5 -n 2 -x 2000. The aligned reads were converted to WIG files using custom scripts available on request. WIG files were normalized to 10 million mapped reads. The data have been deposited in the NCBI Gene Expression Omnibus (GEO) database with accession number GSE103695.

### ChIP-seq analysis

The posterior embryo ChIP-seq data were compared with published data on whole embryos from the same developmental stage (Bradley et al. 2010) downloaded from the NCBI Gene Expression Omnibus (GEO) database with accession number GSM511083. To compare against ZLD binding, previously published data (Harrison et al. 2011) were downloaded from the NCBI GEO database with accession number GSM763061. Since the purpose of the analysis was to compare relative enrichment at genomic loci over each data set's respective background, the following normalization procedure was used: First, for each chromosome, the average of the signal over the entire chromosome (a proxy for the background signal) was subtracted; negative values were then treated as below the background and discarded. The data were then normalized to the background-subtracted average of each chromosome such that the normalized data now represent enrichment over background. For visualization, data were smoothed using a Savitzky-Golay filter of order 3 over a 0.250-kb sliding window. For analysis on CRMs, the RedFly annotation database was used. For analysis centered on called peaks in either the whole-embryo BCD data or the ZLD data BED files containing peak locations were downloaded from the NCBI GEO database.

## Supplementary Material

Supplemental Material
