# Supplementary material for: Dense Bicoid hubs accentuate binding along the morphogen gradient
Source: Genes Dev. 2017 Sep 1;31(17):1784–94. doi: 10.1101/gad.305078.117 (PMC5666676; doi:10.1101/gad.305078.117)
Supplement: Supplemental Material [file supp_31_17_1784__index.html]

Supplemental Material 

# Dense Bicoid hubs accentuate binding along the morphogen gradient

## Supplemental Material

- Supplemental\_Fig\_S1.pdf
- Supplemental\_Fig\_S2.pdf
- Supplemental\_Fig\_S3.pdf
- Supplemental\_Fig\_S4.pdf
- Supplemental\_Fig\_S5.pdf
- Supplemental\_Fig\_S6.pdf
- Supplemental\_Fig\_S7.pdf
- Supplemental\_Fig\_S8.pdf
- Supplemental\_Fig\_S9.pdf
- Supplemental\_Fig\_S10.pdf
- Supplemental\_Fig\_S11.pdf
- Supplemental\_Movie\_S1.avi
- Supplemental\_Movie\_S2.avi
- Supplemental\_Movie\_S3.avi
- Supplemental\_Movie\_S4.avi
- Supplemental\_Table\_S1.pdf
- Supplemental\_Materials\_List.pdf
- Supplemental\_Movies\_Legends.pdf
