# Supplementary material for: Dense Bicoid hubs accentuate binding along the morphogen gradient
Source: Genes Dev. 2017 Sep 1;31(17):1784–94. doi: 10.1101/gad.305078.117 (PMC5666676; doi:10.1101/gad.305078.117)
Supplement: Supplemental Material [file supp_31.17.1784_Supplemental_Fig_S1.pdf]

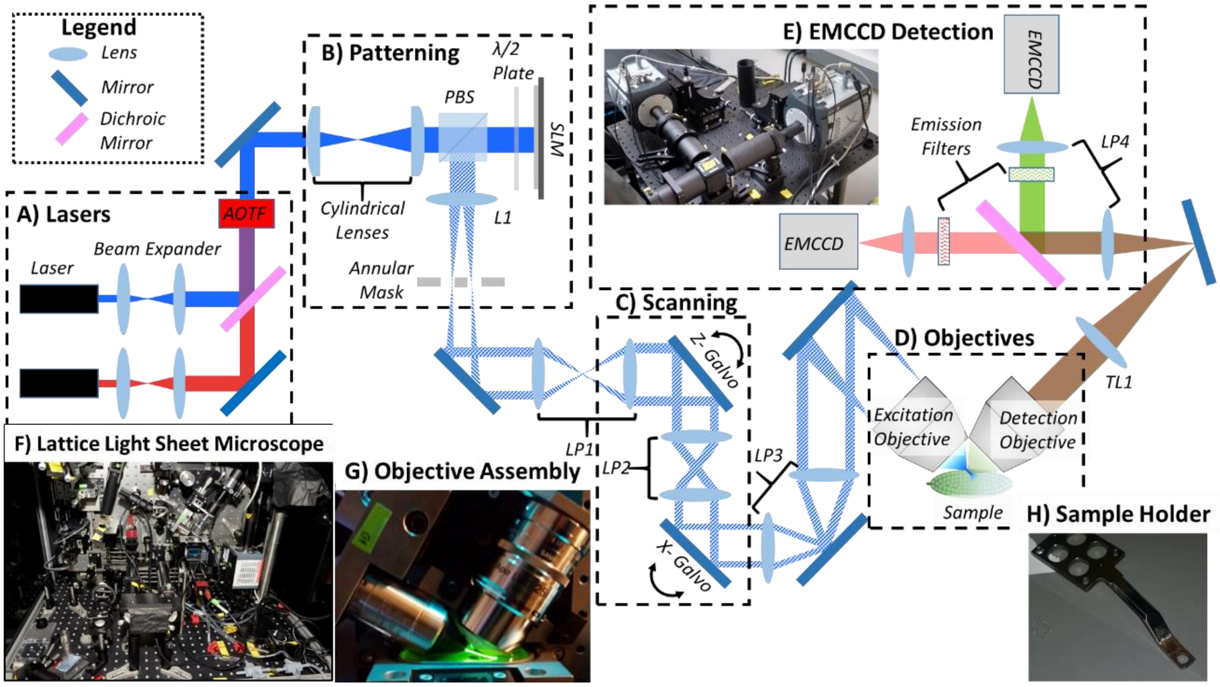


**Supplemental Figure S1. Lattice Light-Sheet Microscope Implementation**

The Lattice light-sheet was built as described by Chen et al, Science, 2014. This simplified schematic shows the major modules of the microscope as follows: **(A)** Laser Module, contains 6 lasers ranging from 405 nm to 639 nm, which are independently expanded, collimated, and input into an Acousto-Optic Tunable Filter (AOTF). **(B)** The patterning module contains a pair of cylindrical lens to expand the input Gaussian beam to a stripe, and a half-wave (λ/2) plate, a polarizing beam splitter (PBS), and a Spatial Light Modulator (SLM) to perform the patterning. Lens L1 projects the Fourier Transform of the SLM pattern onto an annular mask for spatial filtering (**C**) Lens pair 1 (LP1) is then used to de-magnify the annular mask plane and project it onto the z-scan galvo and LP2 projects the z-galvo plane onto the  x-scanning galvo. (**D**) LP3 magnifies the x-galvo plane and projects it onto the back pupil plane of the excitation objective, where it is focused (Fourier transform) to project the final light sheet pattern onto the sample. Emitted fluorescence is collected by the detection objective. (**E**) The tube lens (TL1) focuses an

intermediate image plane which is then magnified using LP4 and projected onto an EMCCD. A dichroic mirror is placed between the first and second lenses of LP4 to split the signal into high and low wavelengths. An emission filter is placed after the dichroic on both sides to select the chromatic bandwidth of the signal reaching each EMMCD independently. Inset shows an image of the EMCCD detection module (**F**) Image of our lattice light sheet microscope. (**G**) Image of the Objective assembly with a dye solution in the sample chamber to visualize the excitation light. (**H**) Image of the sample holder on which the 5 mm coverslip is mounted.
