# Supplementary material for: Dense Bicoid hubs accentuate binding along the morphogen gradient
Source: Genes Dev. 2017 Sep 1;31(17):1784–94. doi: 10.1101/gad.305078.117 (PMC5666676; doi:10.1101/gad.305078.117)
Supplement: Supplemental Material [file supp_31.17.1784_Supplemental_Fig_S2.pdf]

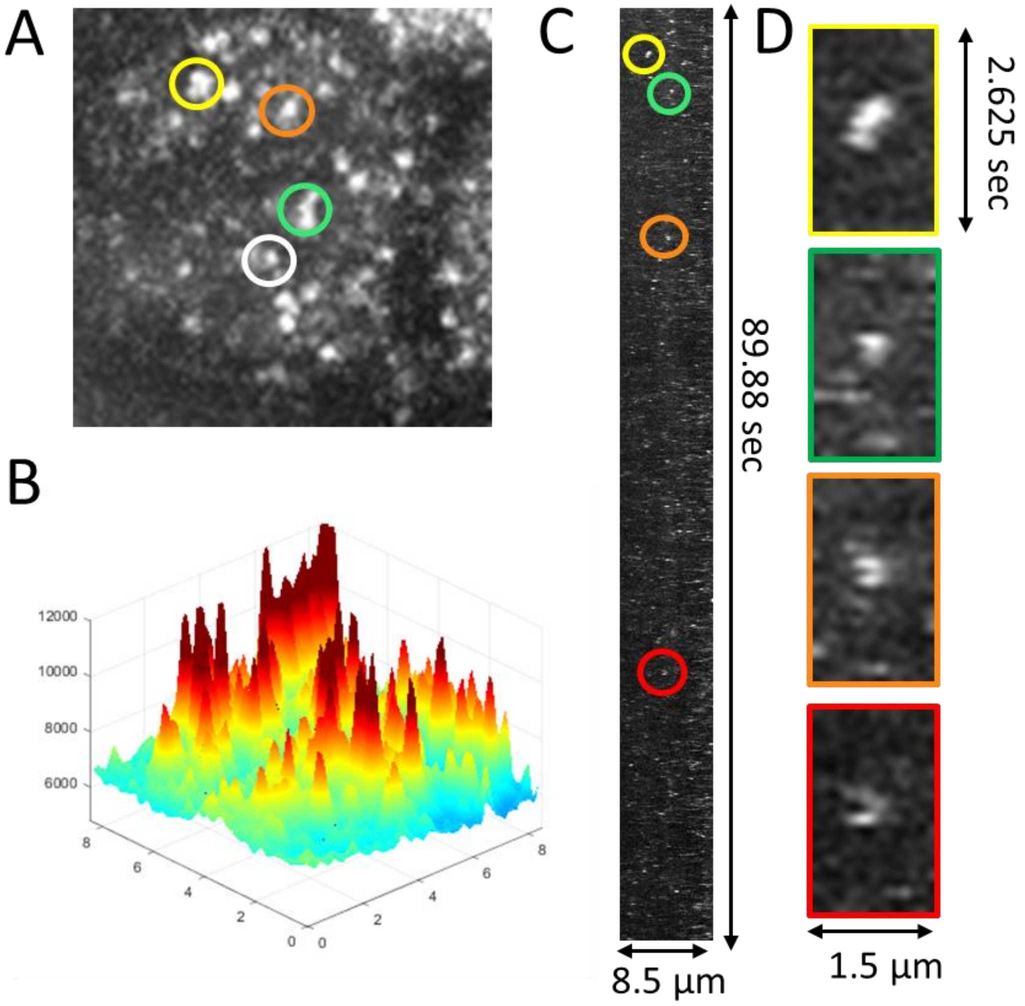

Supplemental Figure S2. **Single Molecule Imaging of BCD-eGFP at 100 milliseconds to estimate residence times**. **(A)** The max projection in time of a 90 second segment of a representative 100 millisecond dataset acquired at an anterior nucleus (EL (x/L) of 0.1), corresponding to the last frame of Video 2. **(B)** Surface plot representation of (A) to illustrate the signal-to-background ratio of single molecule binding events. The smaller peaks likely correspond to slowly diffusing molecules that are not in the imaging volume for the entire exposure time. **(C)** Maximum projection through x-t (kymograph representation) of the data shown in Video 2 from which (A-B) were calculated. Colored circles correspond to those in (A). **(D)** Zoomed in x-t view of the circled regions in (A) and (C), illustrating the transient nature of BCD binding.
