# Supplementary material for: Dense Bicoid hubs accentuate binding along the morphogen gradient
Source: Genes Dev. 2017 Sep 1;31(17):1784–94. doi: 10.1101/gad.305078.117 (PMC5666676; doi:10.1101/gad.305078.117)
Supplement: Supplemental Material [file supp_31.17.1784_Supplemental_Fig_S3.pdf]

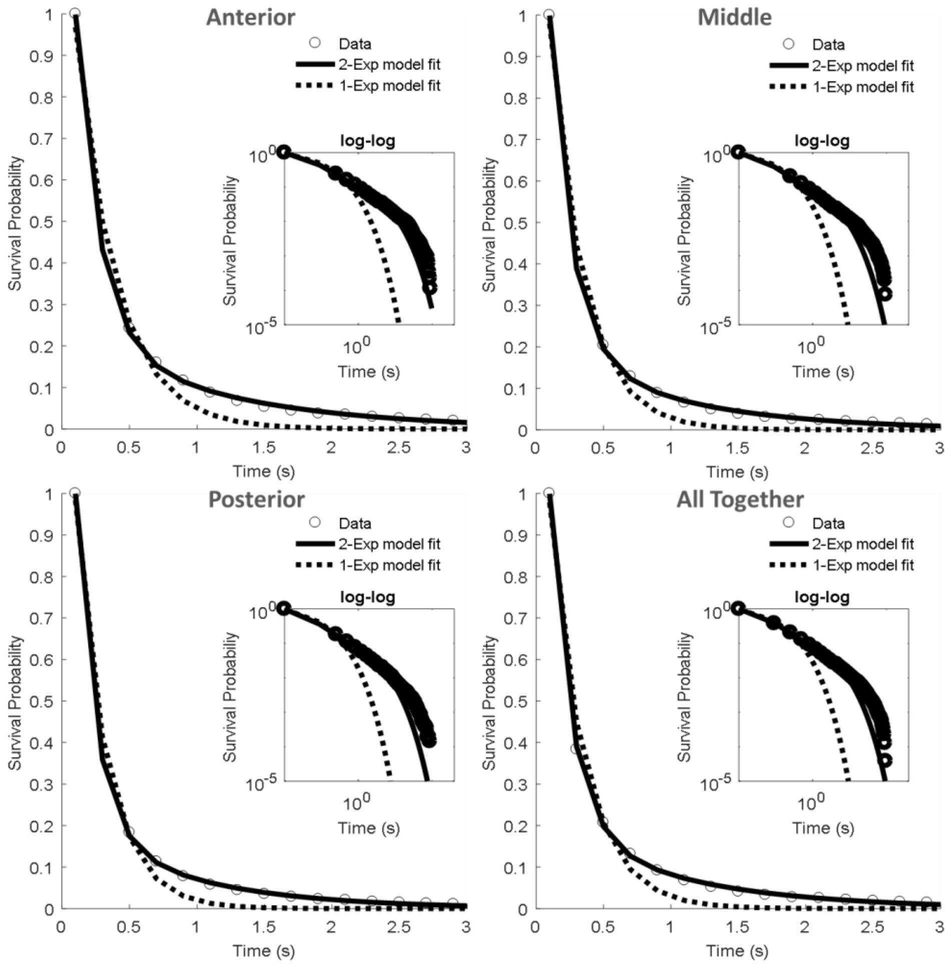

Supplemental Figure S3. Fits to the survival probability distributions of the 100 millisecond datasets. The survival probability distributions calculated from the trajectories output by the MTT analysis on the 100 millisecond data set for the Anterior (34 nuclei, 17735, trajectories), Middle (70 nuclei, 40092 trajectories), Posterior (83 nuclei; 20823 trajectories) segments and all the data pooled together (187 nuclei, 78650 trajectories). The solid lines show the fit to a 2 exponent-model and the dashed lines to a 1-exponent model. Insets show the same on a log-log scale to visualize the difference between 1 and 2 exponent fits at longer time scales. All two-exponent fits have an R^2 value>0.99. See Table 1 for a summary of the fit parameters.
