# Supplementary material for: Dense Bicoid hubs accentuate binding along the morphogen gradient
Source: Genes Dev. 2017 Sep 1;31(17):1784–94. doi: 10.1101/gad.305078.117 (PMC5666676; doi:10.1101/gad.305078.117)
Supplement: Supplemental Material [file supp_31.17.1784_Supplemental_Fig_S4.pdf]

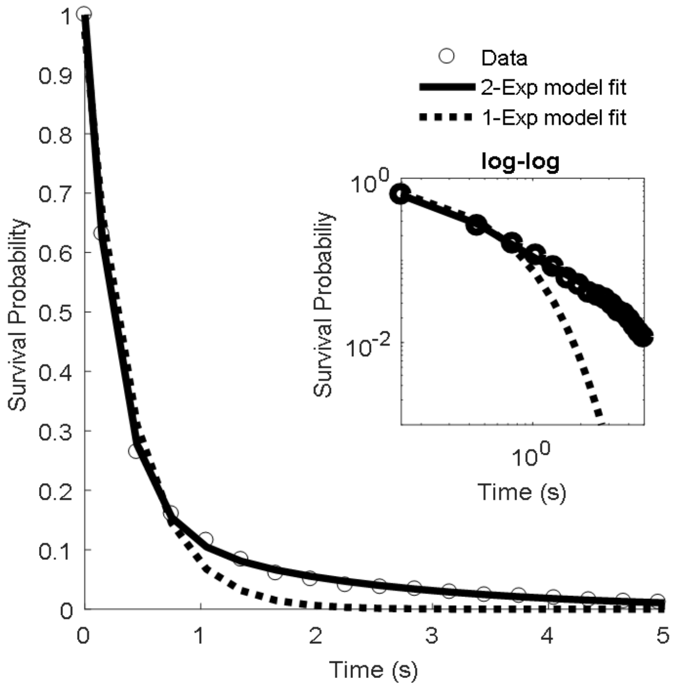

Supplemental Figure S4. Fits to the survival probability distribution of the 500 millisecond dataset. The survival probability distribution of residence times calculated from the MTT algorithm on the 500 ms data set of 17 nuclei and 1211 trajectories. The solid lines show the fit to a 2-exponent model and the dotted lines to a 1-exponent model. The insets shows the same on a log-log scale to visualize the difference between 1 and 2 exponent fits. R^2 value for the 2-exponent fit is 0.99
