# Supplementary material for: Dense Bicoid hubs accentuate binding along the morphogen gradient
Source: Genes Dev. 2017 Sep 1;31(17):1784–94. doi: 10.1101/gad.305078.117 (PMC5666676; doi:10.1101/gad.305078.117)
Supplement: Supplemental Material [file supp_31.17.1784_Supplemental_Fig_S5.pdf]

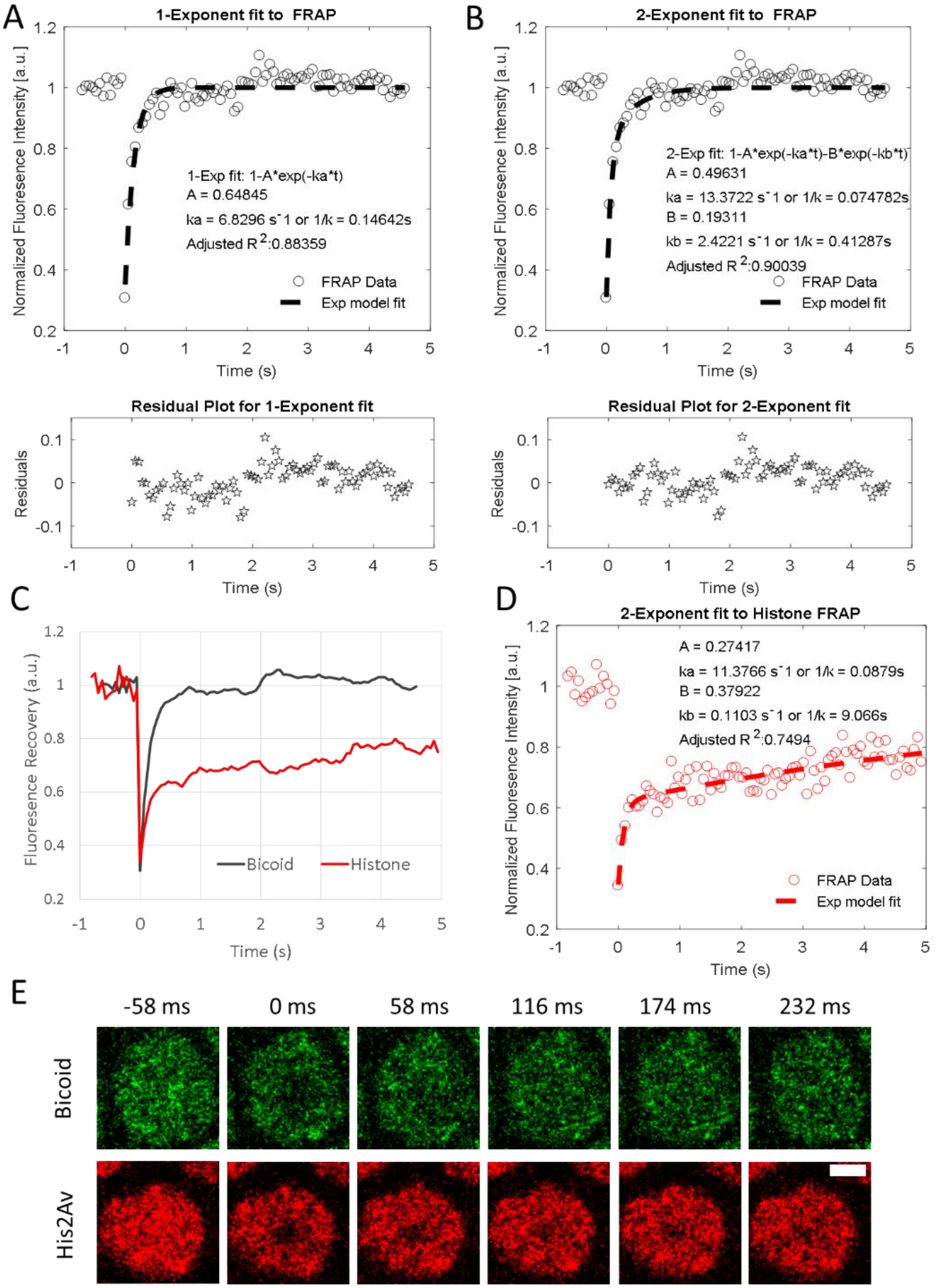

Supplemental Figure S5. Analysis of FRAP data

(A) Averaged Bcd-eGFP FRAP data and single exponential fit results**. (B)** Double exponential fit results to average Bcd-eGFP FRAP**. (C)** Comparison of averaged BCD-eGFP (21 nuclei) and

His2AV FRAP data (3 nuclei). **(D)** Double exponential fit to Histone (His2AV) FRAP. **(E)** Representative images from BCD-eGFP and His2AV FRAP experiments, white scale bar is 2µm.
