# Supplementary material for: Dense Bicoid hubs accentuate binding along the morphogen gradient
Source: Genes Dev. 2017 Sep 1;31(17):1784–94. doi: 10.1101/gad.305078.117 (PMC5666676; doi:10.1101/gad.305078.117)
Supplement: Supplemental Material [file supp_31.17.1784_Supplemental_Fig_S6.pdf]

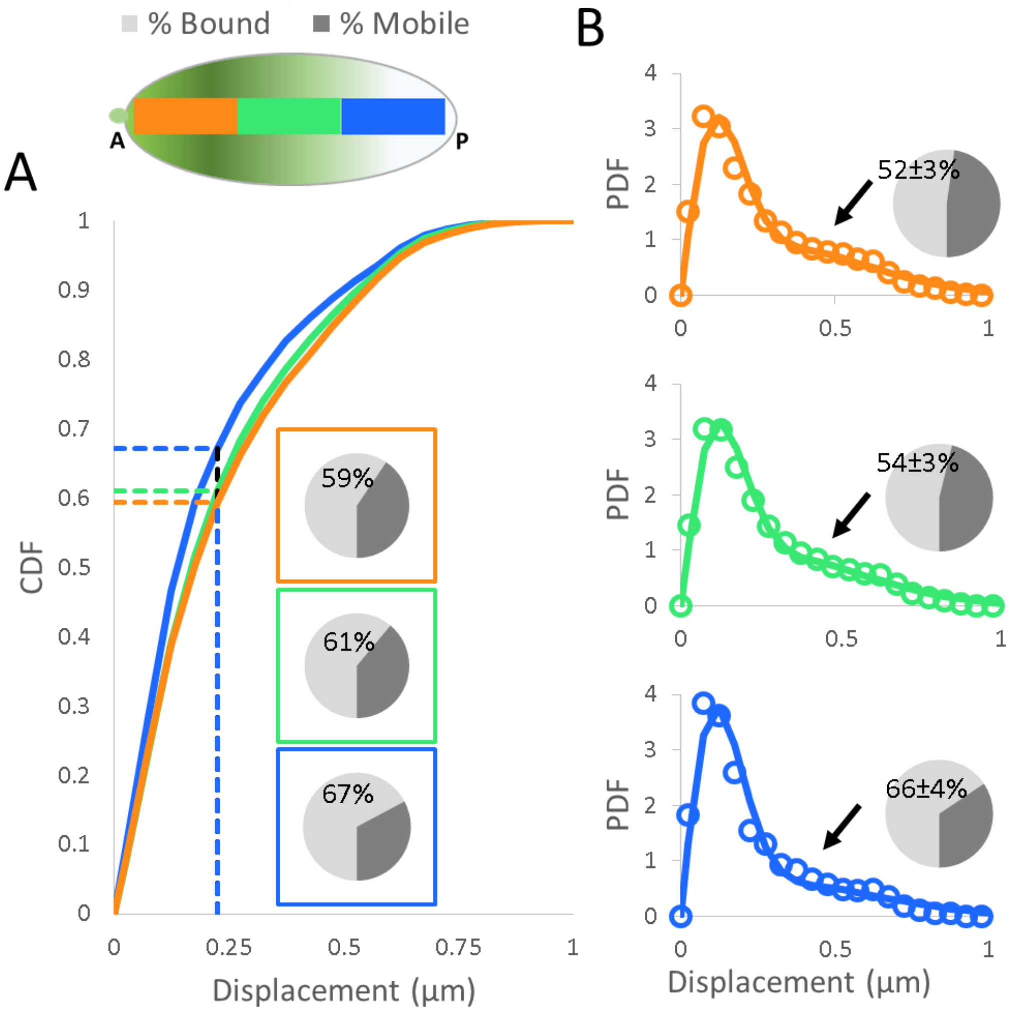

Supplemental Figure S6. Analysis of Displacement Distributions (A) Cumulative distribution functions of the displacement data, pie charts indicate the Bound and Mobile fraction as measured by the CDF value at 0.225 μm (dashed lines). (B) Probability Density Function of the displacement data (markers) and fits to the two population model (solid lines), pie charts show the mobile and bound fractions from the results of the fit, labels indicate the bound fraction and the error is the standard error associated with the fit parameter. Arrows point to the mobile population in the distributions that is decreasing from anterior to posterior positions. (A-B) For each position the

following number of nuclei and trajectories were included in the analysis Anterior: 30 nuclei, 12923, trajectories, Middle: 67 nuclei, 23640 trajectories, Posterior: 66 nuclei; 8600 trajectories.
