# Supplementary material for: Dense Bicoid hubs accentuate binding along the morphogen gradient
Source: Genes Dev. 2017 Sep 1;31(17):1784–94. doi: 10.1101/gad.305078.117 (PMC5666676; doi:10.1101/gad.305078.117)
Supplement: Supplemental Material [file supp_31.17.1784_Supplemental_Fig_S7.pdf]

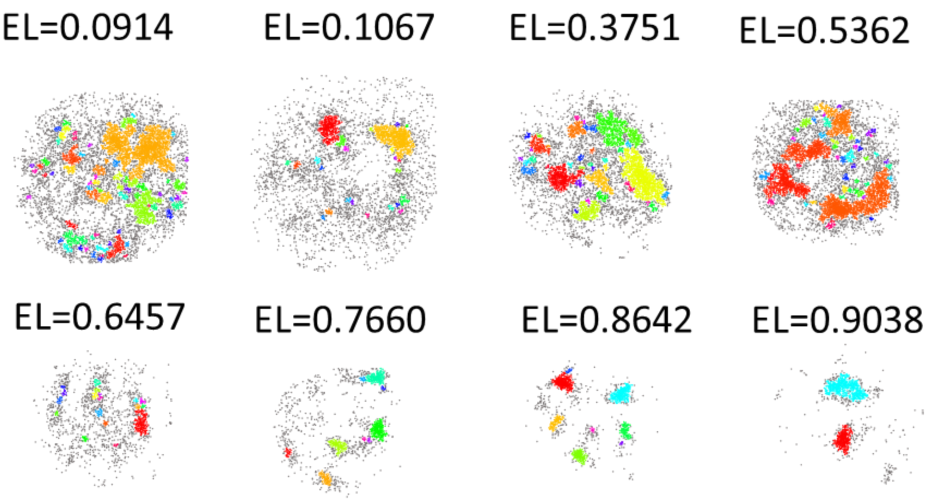

Supplemental Figure S7. Cluster identification results from DBSCAN across the A-P axis. Examples of clusters identified along the A-P axis using DBSCAN with the position shown as fraction of embryonic length (EL). Particles included in the same clusters are represented with the same color.
