# Supplementary material for: Dense Bicoid hubs accentuate binding along the morphogen gradient
Source: Genes Dev. 2017 Sep 1;31(17):1784–94. doi: 10.1101/gad.305078.117 (PMC5666676; doi:10.1101/gad.305078.117)
Supplement: Supplemental Material [file supp_31.17.1784_Supplemental_Fig_S8.pdf]

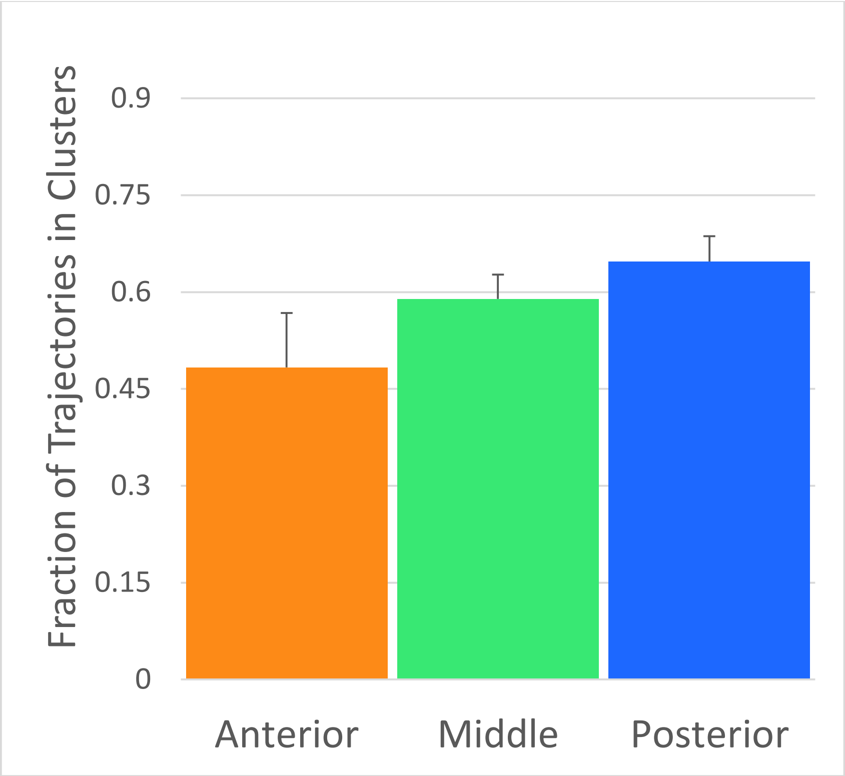

Supplemental Figure S8. Fraction of Trajectories within clusters across the A-P axis. Calculated as trajectories within clusters over total number of trajectories in each nucleus. The median for all nuclei at each spatial position is shown, error bars show standard error.
