# Supplementary material for: Dense Bicoid hubs accentuate binding along the morphogen gradient
Source: Genes Dev. 2017 Sep 1;31(17):1784–94. doi: 10.1101/gad.305078.117 (PMC5666676; doi:10.1101/gad.305078.117)
Supplement: Supplemental Material [file supp_31.17.1784_Supplemental_Fig_S9.pdf]

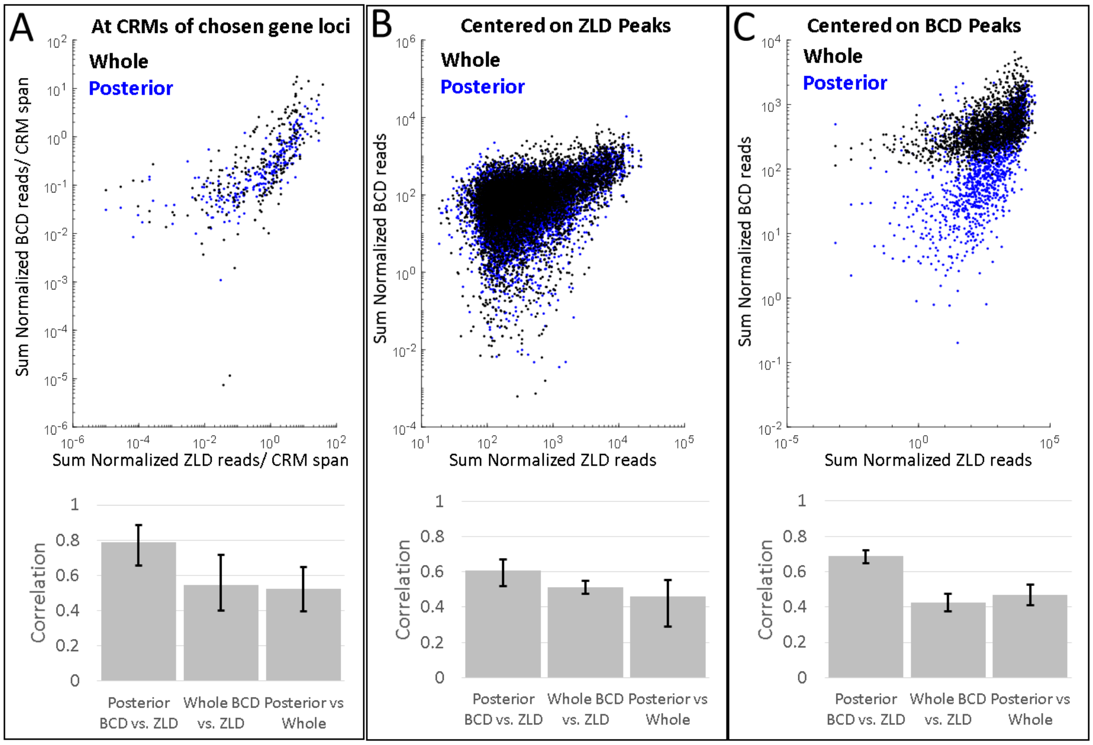

Supplemental Figure S9. BCD binding in whole and posterior thirds embryos compared to ZLD binding. (A) Sum of Normalized BCD ChIP-seq reads divided by the length of the respective CRMs for posterior thirds (blue) and whole embryo (black) vs. Sum of Normalized ZLD ChIP-seq reads divided by the length of the respective CRM at annotated cis-regulatory modules (CRMs) of *eve*, *giant*, *hunchback*, *knirps*, *hairy*, *kruppel*, *caudal*, *fushi-tarazu*, *engrailed*, *wingless*, *runt*, and *gooseberry* loci. A total of 293 CRMs from the RedFly database were analyzed. **(A-B)** Sum of Normalized BCD ChIP-seq reads for posterior thirds (blue) and whole embryo (black) vs. Sum of Normalized ZLD ChIP-seq reads over a 500 bp region centered on **(B)** 8331 ZLD peaks and **(C)** 2145 BCD peaks. **(A-C)** Corresponding bar plots show Pearson correlation coefficients and error bars show 95% confidence intervals as determined by bootstrapping.
