# Supplementary material for: Dense Bicoid hubs accentuate binding along the morphogen gradient
Source: Genes Dev. 2017 Sep 1;31(17):1784–94. doi: 10.1101/gad.305078.117 (PMC5666676; doi:10.1101/gad.305078.117)
Supplement: Supplemental Material [file supp_31.17.1784_Supplemental_Fig_S10.pdf]

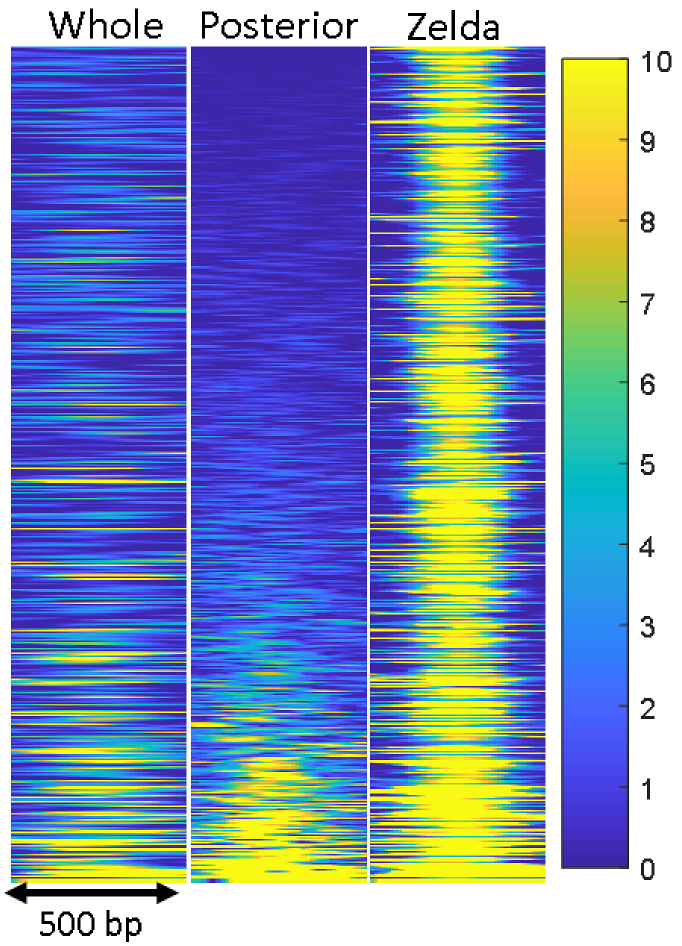

Supplemental Figure S10. BCD and ZLD binding at Zelda peaks. Heat-map representation of normalized ChIP-seq reads for BCD (1st two panels) and ZLD (3rd panel) in a 500 bp window centered on ZLD peaks sorted according to increasing BCD signal in the posterior embryo data. A total of 8331 peaks are shown.
