# Supplementary material for: Dense Bicoid hubs accentuate binding along the morphogen gradient
Source: Genes Dev. 2017 Sep 1;31(17):1784–94. doi: 10.1101/gad.305078.117 (PMC5666676; doi:10.1101/gad.305078.117)
Supplement: Supplemental Material [file supp_31.17.1784_Supplemental_Fig_S11.pdf]

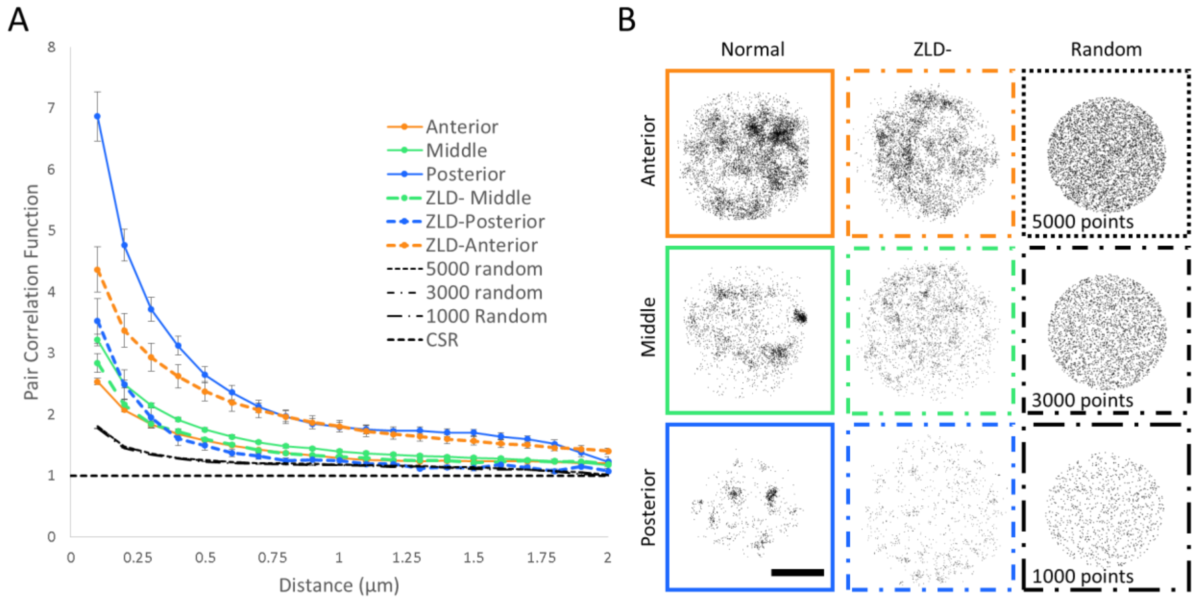

Supplemental Figure S11. Averaged pair-correlation (radial distribution) functions for nuclei in the normal, ZLD- and simulated cases and representative images. (A) Pair-correlation functions. Error bars indicate the standard error. **(B)** Representative images corresponding to pair-correlation functions for Normal, ZLD- and randomly distributed points.
