# Supplementary material for: Dense Bicoid hubs accentuate binding along the morphogen gradient
Source: Genes Dev. 2017 Sep 1;31(17):1784–94. doi: 10.1101/gad.305078.117 (PMC5666676; doi:10.1101/gad.305078.117)
Supplement: Supplemental Material [file supp_31.17.1784_Supplemental_Table_S1.pdf]

Data Set	A-P Poistion	Non-specific binding time ($1/k_{ns}$ (sec))	Specific Binding time ($1/k_s$ (sec))	Corrected Specific Binding time $1/(k_s-k_{bleach})$ (sec)	Number of trajectories
100 ms	Ant	0.175 *[0.166, 0.184]*	1.100 *[1.013, 1.203]*	1.105	17735
	Mid	0.160 *[0.152,0.168]*	0.940 *[0.859, 1.038]*	0.944	40092
	Post	0.146 *[0.141, 0.151]*	0.865 *[0.806, 0.932]*	0.868	20823
	All Together	0.159 *[0.166, 0.152]*	0.959 *[0.882, 1.050]*	0.963	78650
100 ms (Zelda-)	Ant	0.152 *[0.146, 0.160]*	0.777 *[0.726,0.836]*	0.780	11415
	Mid	0.131 *[0.126, 0.137]*	0.629 *[0.587, 0.679]*	0.631	7572
	Post	0.112 *[0.108, 0.117]*	0.463 *[0.435, 0.495]*	0.464	3606
	All Together	0.139 *[0.133, 0.145]*	0.694 *[0.647, 0.748]*	0.696	22593
500 ms	All Together	0.265 *[0.255, 0.277]*	1.876 *[1.69, 2.107]*	-	1211

Supplemental Table S1. Results from 2-exponent model fits to survival probability distributions. k_{ns} and k_s are the un-corrected off-rates for the short-lived (non-specific) and longer-lived (specific) populations respectively determined from a two-exponent fits to the survival probability distributions. The binding time (one-over the off rates) are shown with 95% confidence intervals in square brackets. The photo-bleaching corrected binding times are calculated as $1/(k_s-k_{bleach})$ where k_{bleach} is 0.0043 s^{-1}).
