# Supplementary material for: Dense Bicoid hubs accentuate binding along the morphogen gradient
Source: Genes Dev. 2017 Sep 1;31(17):1784–94. doi: 10.1101/gad.305078.117 (PMC5666676; doi:10.1101/gad.305078.117)
Supplement: Supplemental Material [file supp_31.17.1784_Supplemental_Materials_List.pdf]

LIST of SUPPLEMENTARY MATERIALS

Mir et al., Dense Bicoid Hubs Accentuate Binding along the Morphogen Gradient

Supplemental Movie S1 (Related to Figure 1). Movies corresponding to the still frames shown in Figure 1A.

Supplemental Movie S2 (Related to Figure 1) Representative data from a 90 second segment of a 100 millisecond exposure time movie acquired at an anterior position (EL (x/L) of 0.1). Top left shows the raw data and top right the corresponding surface plot representation. Bottom left shows a running max projection of the data and bottom right shows a surface plot representation of the same

Supplemental Movie S3 (Related to Figure 2) Representative data acquired at 10 millisecond exposure times for 4 nuclei.

Supplemental Movie S4 (Related to Figures 2 and 3) Temporal dynamics of cluster formation for representative nuclei at Anterior, Middle, and Posterior positions.

Supplemental Table S1 (Related to Figures 1 and 3). Results from 2-exponent model fits to survival probability distributions.

Supplemental Fig. S1 (Related to Figure 1). Lattice Light-Sheet Microscope Implementation

Supplemental Fig. S2 (Related to Figure 1). Single Molecule Imaging of BCD-eGFP at 100 milliseconds to estimate residence times.

Supplemental Fig. S3 (Related to Figure 1). Fits to the survival probability distributions of the 100 millisecond datasets

Supplemental Fig. S4 (Related to Figure 1). Fits to the survival probability distribution of the 500 millisecond dataset

Supplemental Fig. S5 (Related to Figure 1). Analysis of FRAP data

Supplemental Fig. S6 (Related to Figure 2). Analysis of Displacement Distributions

Supplemental Fig. S7 (Related to Figure 2). Cluster identification results from DBSCAN across the A-P axis.

Supplemental Fig. S8 (Related to Figure 2). Fraction of trajectories within clusters across the A-P axis.

Supplemental Fig. S9 (Related to Figure 3)**.** BCD binding in whole and posterior thirds embryos compared to ZLD binding

Supplemental Fig. S10 (Related to Figure 3)**.** BCD and ZLD binding at Zelda peaks

Supplemental Fig. S11 (Related to Figures 2 and 3)**.** Averaged pair-correlation (radial distribution) functions for nuclei in the WT, ZLD- , and simulated cases and representative images.
