# Supplementary material for: Dense Bicoid hubs accentuate binding along the morphogen gradient
Source: Genes Dev. 2017 Sep 1;31(17):1784–94. doi: 10.1101/gad.305078.117 (PMC5666676; doi:10.1101/gad.305078.117)
Supplement: Supplemental Material [file supp_31.17.1784_Supplemental_Movies_Legends.pdf]

Captions for Supplemental Movies

Mir et al., Dense Bicoid Hubs Accentuate Binding along the Morphogen Gradient

Supplemental Movie S1 (Related to Figure 1). Movies corresponding to the still frames shown in Figure 1A.

Supplemental Movie S2 (Related to Figure 1) Representative data from a 90 second segment of a 100 millisecond exposure time movie acquired at an anterior position (EL (x/L) of 0.1). Top left shows the raw data and top right the corresponding surface plot representation. Bottom left shows a running max projection of the data and bottom right shows a surface plot representation of the same

Supplemental Movie S3 (Related to Figure 2) Representative data acquired at 10 millisecond exposure times for 4 nuclei.

Supplemental Movie S4 (Related to Figures 2 and 3) Temporal dynamics of cluster formation for representative nuclei at Anterior, Middle, and Posterior positions.
